# Helical domain of hGBP3 cannot stimulate the second phosphate cleavage of GTP

**DOI:** 10.1016/j.jbc.2024.105696

**Published:** 2024-01-30

**Authors:** Divya Rashmi, Sowmiya Gupta, Tasneem Kausar, Apurba Kumar Sau

**Affiliations:** Protein Engineering Laboratory, National Institute of Immunology, New Delhi, India

**Keywords:** large GTPases, human guanylate bining proteins, conformational changes, MD simulations, fluorescence

## Abstract

Interferon-gamma-inducible large GTPases, hGBPs, possess antipathogenic and antitumor activities in human cells. Like hGBP1, its closest homolog, hGBP3 has two domains; an N-terminal catalytic domain and a C-terminal helical domain, connected by an intermediate region. The biochemical function of this protein and the role of its domains in substrate hydrolysis have not yet been investigated. Here, we report that while hGBP3 can produce both GDP and GMP, GMP is the minor product, 30% (unlike 85% in hGBP1), indicating that hGBP3 is unable to produce enhanced GMP. To understand which domain(s) are responsible for this deficiency, we created hGBP3 truncated variants. Surprisingly, GMP production was similar upon deletion of the helical domain, suggesting that in contrast to hGBP1, the helical domain of hGBP3 cannot stimulate the second phosphate cleavage of GTP. We conducted computational and solution studies to understand the underlying basis. We found that the regulatory residue W79, present in the catalytic domain, forms an H-bond with the backbone carbonyl of K76 (located in the catalytic loop) of the substrate-bound hGBP3. However, after gamma-phosphate cleavage of GTP, the W79-containing region does not undergo a conformational change, failing to redirect the catalytic loop toward the beta-phosphate. This is necessary for efficient GMP formation because hGBP homologs utilize the same catalytic residue for both phosphate cleavages. We suggest that the lack of specific interdomain contacts mediated by the helical domain prevents the catalytic loop movement, resulting in reduced GMP formation. These findings may provide insight into how hGBP3 contributes to immunity.

The human guanylate-binding proteins (GBPs) are a group of interferon-gamma-inducible large GTPases (∼65 kDa) that are involved in the innate immune response to intracellular pathogens. Unlike other large GTPases, human homologs of GBP possess the distinct ability to catalyze the hydrolysis of GTP to GDP and further to GMP *via* consecutive phosphate cleavages, resulting in a combination of GDP and GMP (1). Seven human GBP homologs (hGBP1-7) with sequence identities ranging from 75 to 87% are now known (2). These proteins show varying amounts of GMP formation even though they share a high sequence identity. For instance, hGBP1 exhibits substantial GMP production, constituting 85% of the total product (1). On the other hand, hGBP2 produces a significantly lower GMP (5%), and GMP formation in hGBP5 was not detected (3, 4). It is unknown whether other hGBP homologs hydrolyze GTP.

hGBPs show a vast range of effects against different organisms. For instance, hGBP1 exhibits the antiviral response against hepatitis C, dengue, encephalomyocarditis, and vesicular stomatitis (5, 6, 7). Several viruses, including HIV-1, influenza A, murine leukemia, Zika virus, and measles, are inhibited by hGBP2 and hGBP5 (8). Additionally, hGBP1 has antiparasitic and antibacterial properties against *Toxoplasma gondii*, *Shigella flexneri*, and *Chlamydia trachomatis*, respectively (9, 10, 11). Moreover, it has been found that these proteins suppress the growth of tumors in a variety of carcinomas, including breast, colorectal, and prostate (12, 13, 14, 15).

It has been demonstrated that the biological roles of hGBP1 are associated with its enzymatic activity. This protein's substrate binding contributes to its anti-influenza function, but its hydrolysis is crucial in preventing the growth of the Kaposi sarcoma virus (16, 17). Although the substrate hydrolysis by this protein was reported to be essential for the anti-viral activity, it was unclear if the second phosphate cleavage exhibited has any role in this function. Recently, it was found that stimulated GMP formation as a result of substrate hydrolysis is crucial for its anti-HCV efficacy (18). While GMP production is not required to restrict *Chlamydia* growth, it is essential for inflammasome activation (19). These findings perhaps explain why this protein has evolved to perform second phosphate cleavage of GTP for host defense.

hGBP1 is an extensively studied protein and can be considered the prototype of the hGBP family. The full-length hGBP1 has been crystallized in the absence and presence of nonhydrolyzable GTP analog, GppNHp (20, 21). The 592-residue long protein has two distinct domains: an N-terminal catalytic domain (1–278) and a C-terminal helical domain (312–592), which are connected by a short intermediate region (279–311). The catalytic domain contains switch I (residues 68–76), switch II (residues 97–104), and guanine cap (residues 238–257) regions. Notably, the catalytic machinery is present in switch I. In the free protein, these three stretches exist as a loop and change their structure after GTP binding which is essential for stimulating GTP hydrolysis. Furthermore, hGBP1 exists as a monomer in the free form but changes its conformation into an extended monomer after GTP binding. Upon first phosphate cleavage, these extended monomers further dimerize, which generates a conformation that enhances GMP formation by stimulating GTPase activity (22, 23, 24).

While extensive research has been conducted on GTP hydrolysis by hGBP1, the underlying mechanism for substrate hydrolysis by this protein was not fully elucidated. It has been recently suggested that hGBP1 and its homolog hGBP2 employ the same catalytic residue to cleave both the gamma and beta phosphates of GTP (25). It was proposed that after the gamma phosphate hydrolysis, the distance between the beta phosphate of GTP and the catalytic residue did not favor the occurrence of the beta phosphate cleavage. Either the nucleotide or the region containing the catalytic residue should move so that the beta phosphate and the catalytic residue are properly positioned to facilitate the second phosphate cleavage. A comparison between the crystal structures of truncated hGBP1^1–317^, devoid of the helical domain with GDP.AlF_4_^−^, and GMP.AlF_4_^−^ (transition state analogs for the first and second phosphate cleavage, respectively), suggested that the nucleotide moves toward the catalytic residue following the first phosphate cleavage. This may aid in the hydrolysis of GTP's second phosphate. However, recent studies on hGBP1 and hGBP2 suggest that the loop containing the catalytic residue moves toward the beta phosphate after the cleavage of the gamma phosphate, which enhances the beta phosphate cleavage leading to stimulated GMP formation (26). The movement of the above catalytic loop happens in hGBP1, but not in hGBP2, which results in differential GMP formation in these two homologs (85% GMP in hGBP1 *versus* 5% GMP in hGBP2) (18, 25, 27). This highlights the importance of the catalytic loop movement for enhanced GMP formation.

hGBP3 is another member of the hGBP family, which shows the highest sequence identity with hGBP1 (∼87%). According to a recent study on primate GBPs, the GBP3 gene is exclusively found in simiiformes and most likely developed through the duplication of the GBP1 gene (28). Based on the classification of the hGBP1 domains, hGBP3 can be divided into the catalytic and helical domains, which are connected by an intermediate region. This protein is highly expressed in the brain, stomach, small intestine, pancreas, kidney, epididymis, placenta, soft tissues, and bone marrow (proteinatlas.org) (29). In lung epithelial cells, overexpression of hGBP1 and hGBP3 was found to reduce influenza A virus titers by 5- to 10-fold (30). Additionally, the anti-influenza activity of these two proteins is dependent on substrate binding. Despite this, it is still unknown whether the next event, which is the substrate hydrolysis, is essential for its anti-influenza action. Thus, it is essential to know if hGBP3 can hydrolyze GTP similar to hGBP1 and if the GTP hydrolysis is regulated differently. If the regulation is different, what could be the underlying basis? Additionally, how the different domains of this protein regulate GTP hydrolysis has not yet been investigated.

The present study demonstrates that while hGBP3 produces both GDP and GMP, the amount of GMP is relatively less. We also showed that, unlike in hGBP1, the helical domain of hGBP3 plays no role in substrate hydrolysis. Using WT and truncated proteins, our findings indicate that the lower amount of GMP production is mainly due to the helical domain variation, which does not form specific interdomain interactions with the catalytic domain. As a result, the regulatory residue W79 present in the catalytic domain does not seem to move toward the solvent after the first phosphate cleavage of GTP, thereby preventing efficient GMP formation. Overall, this study describes the role of the individual domains of hGBP3 in GTP hydrolysis and provides a basis for the decrease in stimulated GMP formation.

## Results

### hGBP3 possesses GTPase activity but produces less GMP

**Figure 1:**
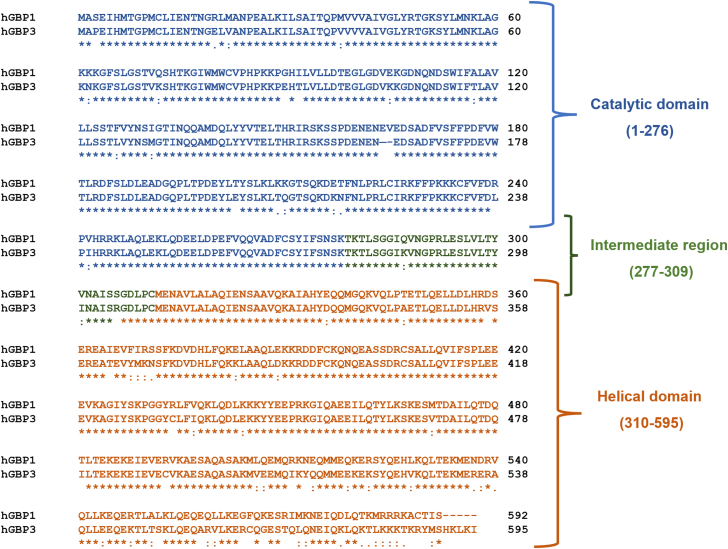
**Sequence alignment of hGBP3 with hGBP1 using Clustal omega.** Based on the structure of hGBP1, hGBP3 is composed of an N-terminal catalytic domain (1–276) and a C-terminal helical domain (310–595), connected with a short intermediate region (277–309). The catalytic domain, intermediate region, and helical domain of these two proteins display 93, 89, and 81% sequence identity, respectively. GBP, guanylate-binding protein.

**Figure 2:**
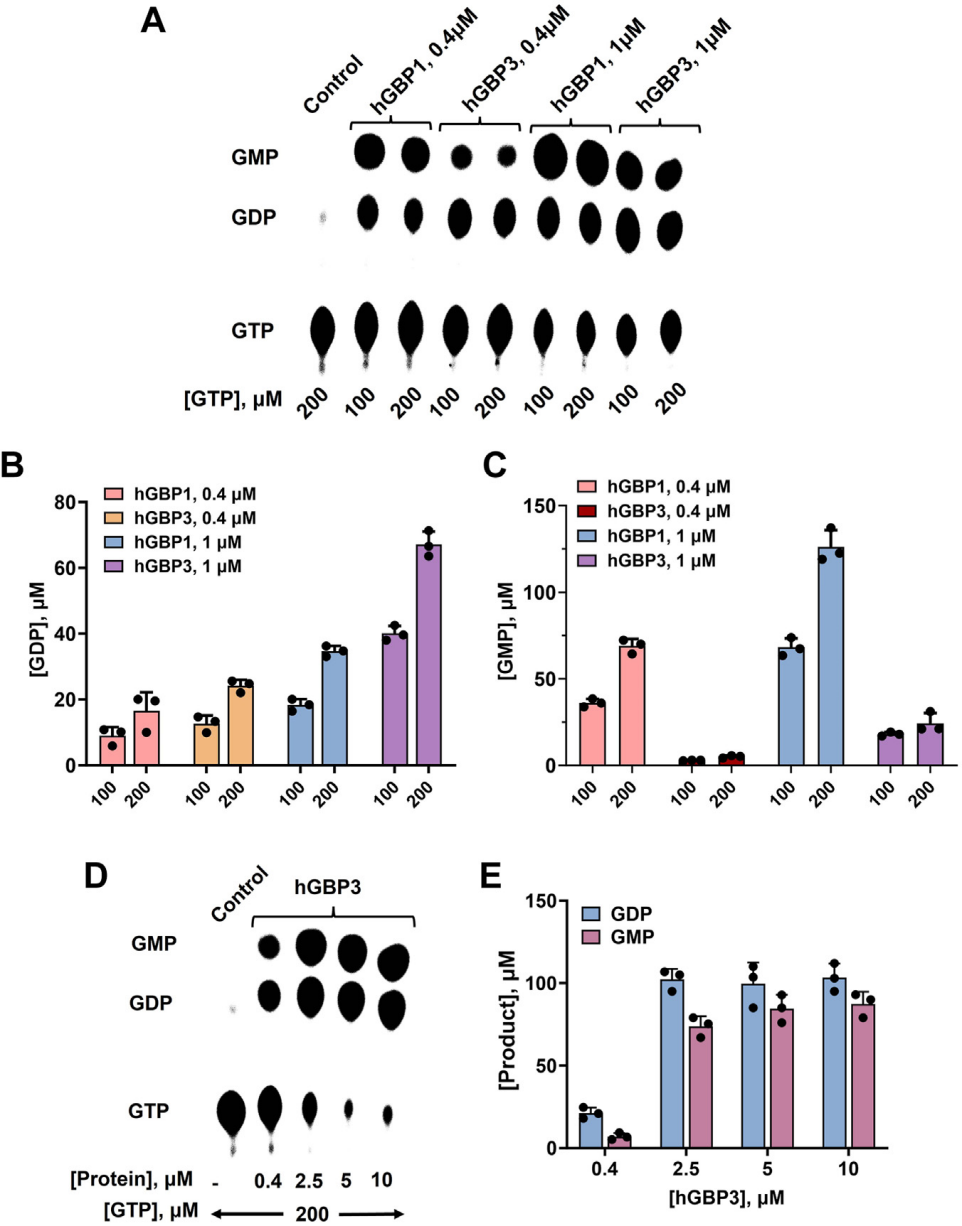
**GTPase activity.***A*, GTPase assay of hGBP3 and hGBP1 at varying substrate and enzyme concentrations. These assays were conducted in a reaction buffer described in the Experimental procedures, utilizing a minute quantity of radiolabeled [α-^32^P] GTP at a temperature of 37 °C. The figure illustrates the specific concentrations of unlabeled GTP, and the enzyme used for each experiment. The lane labeled “control” shows that the experiment was carried out without any enzymes. The figure shown is a representative of one of the assays. *B* and *C*, the bar graphs represent the average GDP and GMP production, respectively, based on the results obtained from the activity assays shown in (*A*). *D*, GTPase assay of hGBP3 at higher protein concentrations using 200 μM unlabeled GTP. *E*, bar graph of the GDP and GMP produced by hGBP3 at higher concentrations. Error bars in each figure correspond to the SDs of three independent experiments. GBP, guanylate-binding protein.

To check if hGBP3 exhibits GTPase activity, we carried out its activity assay using [α-^32^P] radiolabeled GTP. The experiment was performed with a fixed concentration of the protein (0.4 μM) and two different concentrations of unlabeled GTP (100 and 200 μM). We also performed a similar assay with hGBP1 for comparison because hGBP3 shares the highest sequence identity with this protein (87%) among the hGBP homologs. We found that hGBP3 hydrolyzes GTP into GDP as well as GMP similar to hGBP1. However, the GMP formation in hGBP3 was considerably lower than that of hGBP1 at both substrate concentrations (∼5.5-fold less), whereas GDP was almost 2.5-fold higher (Fig. 2, *A*–*C*). This resulted in a significant decrease in the GMP to GDP ratio in hGBP3 as compared to that in hGBP1 (∼0.25:1 for hGBP3 *versus* ∼3.9:1 for hGBP1). The GMP to GDP ratio for hGBP1 is in agreement with the reported data (31). The lower GMP formation in hGBP3 in comparison to hGBP1 suggests that hGBP3 is unable to produce high GMP levels. This could be due to the variation in its helical domain since the helical domain of hGBP1 is known to simulate GTPase activity leading to enhanced GMP production (31). It should be noted that the catalytic residue(s) of hGBP1 present in the N-terminal domain is conserved in hGBP3 and that the helical domain of these two proteins exhibits relatively low sequence identity (∼81% sequence identity in the helical domain *versus* ∼93% sequence identity in the catalytic domain, Fig. 1).

We also performed the activity assay of hGBP3 at a higher protein concentration (1 μM) to check whether the product formation is increased which may lead to the increased GMP to GDP ratio similar to hGBP1. We found that GMP production at this protein concentration was nearly 4-fold lower than that of hGBP1 (Fig. 2, *A* and *B*). This suggests that GMP formation in hGBP3 is comparatively increased at the higher protein concentration. GDP formation was also relatively increased (Fig. 2, *A* and *C*). This has increased the GMP to GDP ratio as compared to that at 0.4 μM hGBP3 protein concentration (from 0.25:1 to 0.42:1). However, the product ratio in hGBP1 at 1 μM protein concentration did not increase (∼3.9:1). This indicates that even at higher protein concentration, hGBP3 cannot increase GMP formation, which is consistent with the previous experiment.

We also performed another activity assay where the substrate concentration was fixed but the concentration of hGBP3 was systematically increased to 10 μM to check whether the above product ratio was increased. Both GDP and GMP at 2.5 μM protein concentration were increased compared to that of 1 μM protein (Fig. 2, *D* and *E*). This has increased the GMP to GDP ratio to some extent (from 0.42:1 to 0.9:1) but still, it is lower than that of hGBP1 at 0.4 and 1 μM protein concentrations. The product formation did not increase further at higher hGBP3 concentrations (5 and 10 μM). This observation again suggests that hGBP3 is unable to generate enhanced GMP. The increase in GMP formation at higher protein concentrations could be due to an increase in the substrate-induced dimerization/oligomerization coefficient of the protein.

### The lower GMP formation is associated with insignificant allosteric regulation

**Figure 3:**
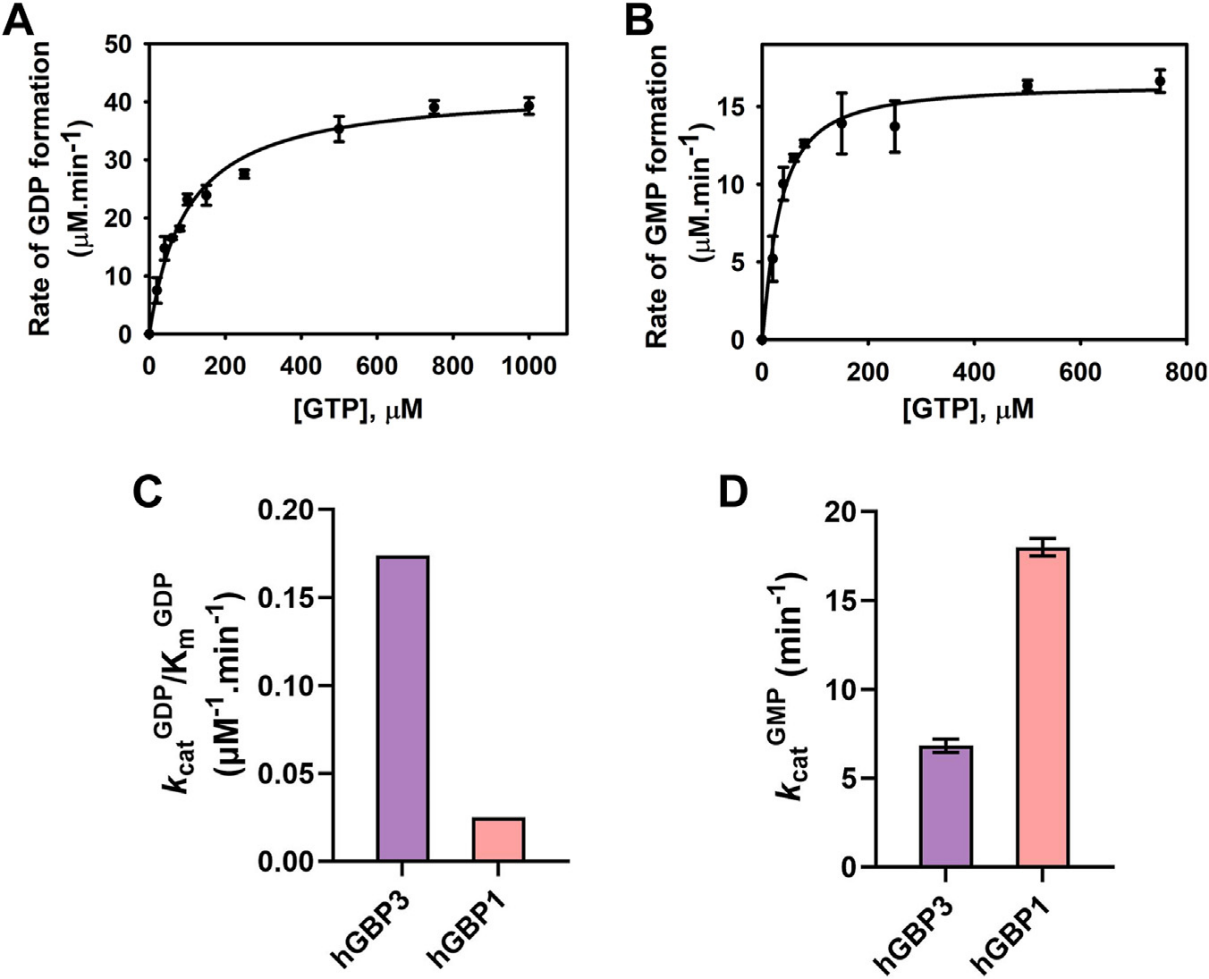
**Steady-state kinetics of hGBP3.** The experiment was carried out with 2.5 μM protein and a minute quantity of radiolabeled [α-^32^P] GTP but with varying amounts of unlabeled GTP as shown in the figure. The initial rates for each product were calculated and plotted against the concentrations of GTP. The quality of the fit was assessed by drawing a theoretical line through the experimental data points and establishing confidence limits. *A*, the plot represents the rate of GDP formation *versus* GTP concentration. The data points were fitted to a hyperbolic equation to determine the *k*_cat_ and K_m_. *B*, plot representing the rate of GMP formation *versus* GTP concentration. The data were fitted using the Hill equation. This allowed the determination of K_m_ (apparent affinity), *k*_cat_ (catalytic constant), and *n* (Hill coefficient). *C* and *D*, the bar graphs represent the kinetic parameters for GDP and GMP formation, respectively, of hGBP3 and hGBP1. The values for hGBP3 are derived from the above plots. The parameters for hGBP1 have been calculated from separate experiments. The errors associated with *k*_cat_ represent the SE derived from the data fitting. But, for the ratio of *k*_cat_ and K_m_, the values are shown without error. GBP, guanylate-binding protein.

To support the activity assay results and obtain insight into the mechanism of GTP hydrolysis, we conducted steady-state kinetic experiments of hGBP3. In these experiments, a fixed concentration of the enzyme was used while the substrate concentrations were systematically increased. To study multiple enzyme turnover kinetics, the substrate was kept in excess over the enzyme. We also conducted a similar kinetic assay with hGBP1 for comparison. To determine the kinetic parameters, K_m_ (apparent affinity of the substrate to an enzyme) and *k*_cat_ (catalytic constant for product formation) for GDP, the rate of GDP formations *versus* the substrate concentrations were fitted to a hyperbolic equation (Fig. 3*A*). However, for GMP, the data were fitted to the Hill equation (Fig. 3*B*) since the GMP formation in hGBP1 was reported to be associated with cooperativity. As previously mentioned, hGBP3 is a close homolog of hGBP1 (87% sequence identity). Therefore, it may be reasonable to believe that hGBP3 hydrolyzes GTP to GMP through a series of phosphate cleavages, similar to hGBP1. Like hGBP1, for hGBP3, the *k*_cat_/K_m_ (catalytic efficiency) for GDP has been considered as a measure of the efficiency of GDP formation since the hydrolysis of GTP to GDP by this enzyme is a bimolecular reaction. However, because the hydrolysis of GDP to GMP is a unimolecular reaction, the *k*_cat_ (catalytic constant) value is used as a measure of GMP production. The *k*_cat_/K_m_ and *k*_cat_ values for GDP and GMP production, respectively, in hGBP1 were found to be 0.025 ± 0.007 μM^−1^ min^−1^ and 18 ± 0.5 min^−1^ (Fig. S1). Additionally, the *n* value (Hill-coefficient) for GMP production was 1.5 ± 0.2. These hGBP1 kinetic parameters are consistent with the reported values (31). The *k*_cat_/K_m_ value for GDP formation in hGBP3 is 0.174 ± 0.01 μM^−1^.min^−1^, which is nearly 7-fold greater than that of hGBP1 (Fig. 3*C*). On the other hand, the *k*_cat_ for GMP formation is nearly 3-fold lower than that in hGBP1, Figure 3*D* (6.8 ± 0.3 min^−1^ for hGBP3 *versus* 18 ± 0.5 min^−1^ for hGBP1, *i.e.*, 33% of hGBP1 GMP formation). These findings imply that hGBP3 produces GDP with a higher efficiency than hGBP1 but yields GMP at a lower catalytic rate. It is interesting to note that the *n* value for GMP formation in hGBP3 is 1.15 ± 0.15, suggesting that unlike in hGBP1, the beta phosphate cleavage of GTP by hGBP3 is associated with negligible cooperativity. Thus, the hGBP3 kinetic data are consistent with its activity assay results and suggest that less GMP production in this protein might be linked to insignificant allosteric regulation.

It may be noted that the helical domain of hGBP1 allosterically regulates GMP formation through its specific interaction(s) with the catalytic domain (24, 32). As described before, among the two domains of hGBP3 and hGBP1, the helical domain shows more sequence variation. Thus, the lower GMP production in hGBP3 than hGBP1 might be due to the variation of the helical domain.

### The helical domain does not contribute to substrate hydrolysis

**Figure 4:**
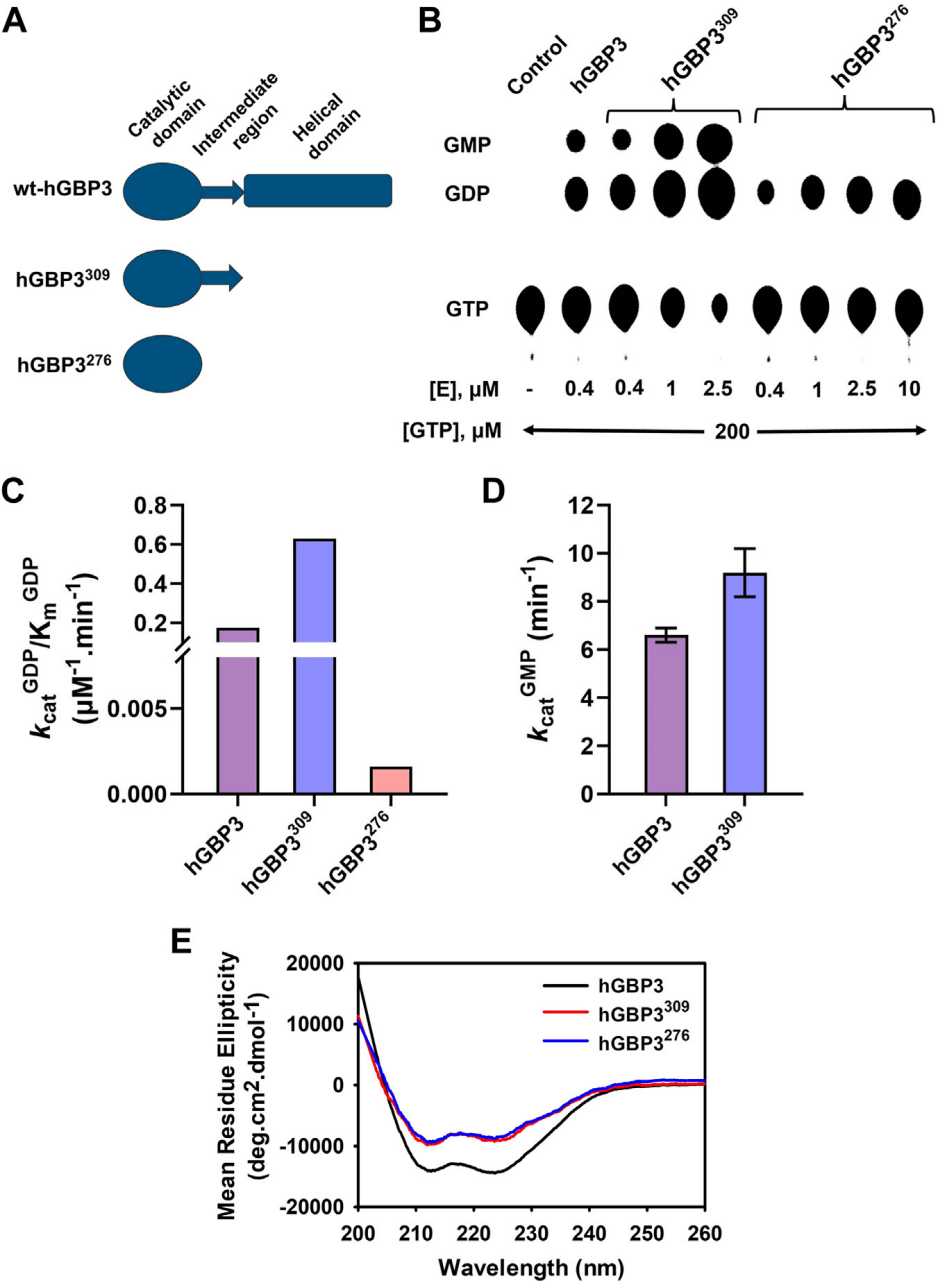
**The role of hGBP3 individual domains in GTP hydrolysis.***A*, a schematic representation of hGBP3 truncated protein constructs. *B*, GTPase assay of hGBP3 and its truncated variants, hGBP3^309^ and hGBP3^276^, at varying enzyme concentrations. The figure displayed here represents the outcome of one of the experiments. *C*, the bar graph represents the specificity constant (*k*_cat_/K_m_) of full-length hGBP3, hGBP3^309^, and hGBP3^276^ for GDP production, derived from the Hill equation. *D*, the catalytic constant (*k*_cat_) values of hGBP3 and hGBP3^309 are^ derived from the hyperbolic equation. The data for hGBP3^309^ and hGBP3^276^ are estimated from the average of three separate experiments. The errors associated with *k*_cat_ represent the SE derived from the data fitting. But, for the ratio of *k*_cat_ and K_m_, the values are shown without error. The kinetic parameters of full-length hGBP3 have been taken from Figure 3. *E*, circular dichroism measurement of hGBP3 and its truncated variants in the far-UV range. The data displayed represent the averages of three independent scans for each protein. GBP, guanylate-binding protein.

Next, to test whether the sequence variation in the helical domain of hGBP3 as compared to hGBP1 is responsible for the lower GMP formation, we prepared a truncated protein construct, hGBP3^309^, in which the helical domain has been deleted (Fig. 4*A*). This truncated protein was overexpressed and purified to homogeneity. Activity assay was performed with increasing protein concentrations, where the substrate concentration was kept at 200 μM. Similar to full-length hGBP3, hGBP3^309^ produced GDP and GMP at 0.4 μM protein concentration (Fig. 4*B*). Both the products in this truncated variant were increased to some extent as compared to that of the full-length protein. Additionally, the product formation was increased with increasing protein concentrations (Fig. S2, *A* and *B*). However, the ratio of GMP to GDP production did not vary. These findings imply that the helical domain of the full-length protein seems to have some negative effect on the product formation, including GMP.

As previously mentioned, the helical domain of hGBP1 stimulates GMP formation. To directly compare the effect of helical domain deletion on GMP formation between hGBP3 and hGBP1, we conducted GTPase assays using the full-length and truncated proteins of hGBP3 and hGBP1. hGBP3^309^ and hGBP1^311^ were used as truncated variants without the helical domains. Unlike in hGBP3, the deletion of the helical domain of hGBP1 significantly reduced GMP production (Fig. S3, *A*–*C*), which is consistent with a previous study (31). GDP formation increased slightly. These results suggest that, in contrast to hGBP1, the helical domain of hGBP3 does not promote GMP production.

Further, to check whether the GMP formation in the truncated protein, hGBP3^309^, is due to the presence of the intermediate region, we prepared another truncated protein construct, hGBP3^276^ in which the intermediate region along with the helical domain was deleted. This truncated variant displayed only GDP. Even at a protein concentration of 10 μM, GMP production could not be seen (Figs. 4*B* and S2*A*). Moreover, this protein produced GDP at a very low level. This finding suggests that the hydrolysis of GTP to just GDP can be accomplished by the catalytic domain alone. However, when this domain and the intermediate region are present together, GDP can be further hydrolyzed to GMP. This implies that the GMP observed in full-length hGBP3 is because of the presence of the intermediate region. It has been reported earlier that dimerization of hGBP1 is essential for GMP formation, which is mediated by the intermediate region. Thus, it is possible that the GMP observed in hGBP3^309^ is due to its intermediate region–mediated dimerization of the protein.

To obtain further insight into the enzyme activity of these two truncated proteins, we performed similar steady-state kinetic experiments. Like full-length hGBP3, the data for GDP and GMP formation in hGBP3^309^ were fitted to the hyperbolic and sigmoidal equations, respectively. The *k*_cat_ value for GMP formation in hGBP3^309^ was 1.4-fold higher than that of the full-length protein (9.3 ± 1 min^−1^ for hGBP3^309^ *versus* 6.8 ± 0.3 min^−1^ for wt-hGBP3, Fig. 4*D*). The *k*_cat_/K_m_ value for GDP production was also increased by ∼3.5-fold (0.63 ± 0.01 μM^−1^.min^−1^ in hGBP3^309^ *versus* 0.17 ± 0.01 μM^−1^.min^−1^ in wt-hGBP3, Fig. 4*C*). Additionally, the *n* value for GMP formation was nearly 0.9 ± 0.2, which is similar to that of the full-length protein. These results suggest that deletion of the helical domain in hGBP3 increases both product formation. This is consistent with the activity assay data and supports our observation that the helical domain of the full-length protein has some negative effect on GTP hydrolysis.

The data for GDP formation in hGBP3^276^ were fitted to a hyperbolic equation, yielding the *k*_cat_/K_m_ value nearly 0.0016 ± 0.001 μM^−1^.min^−1^, which is 110- and 390-fold lower than that of the full-length hGBP3 and hGBP3^309^, respectively (Fig. 4*C*). This suggests that the catalytic domain cannot hydrolyze GTP to GDP efficiently in the absence of the intermediate region. The significantly reduced GDP formation in hGBP3^276^ is comparable to that of small GTPases such as Ras, Rho in the absence of external GTPase-activating protein, as these proteins are known to hydrolyze GTP very slowly to GDP without their accessory proteins. Thus, the intermediate region of hGBP3 can be considered as an internal GTPase-activating protein when it is present along with the catalytic domain.

**Figure 5:**
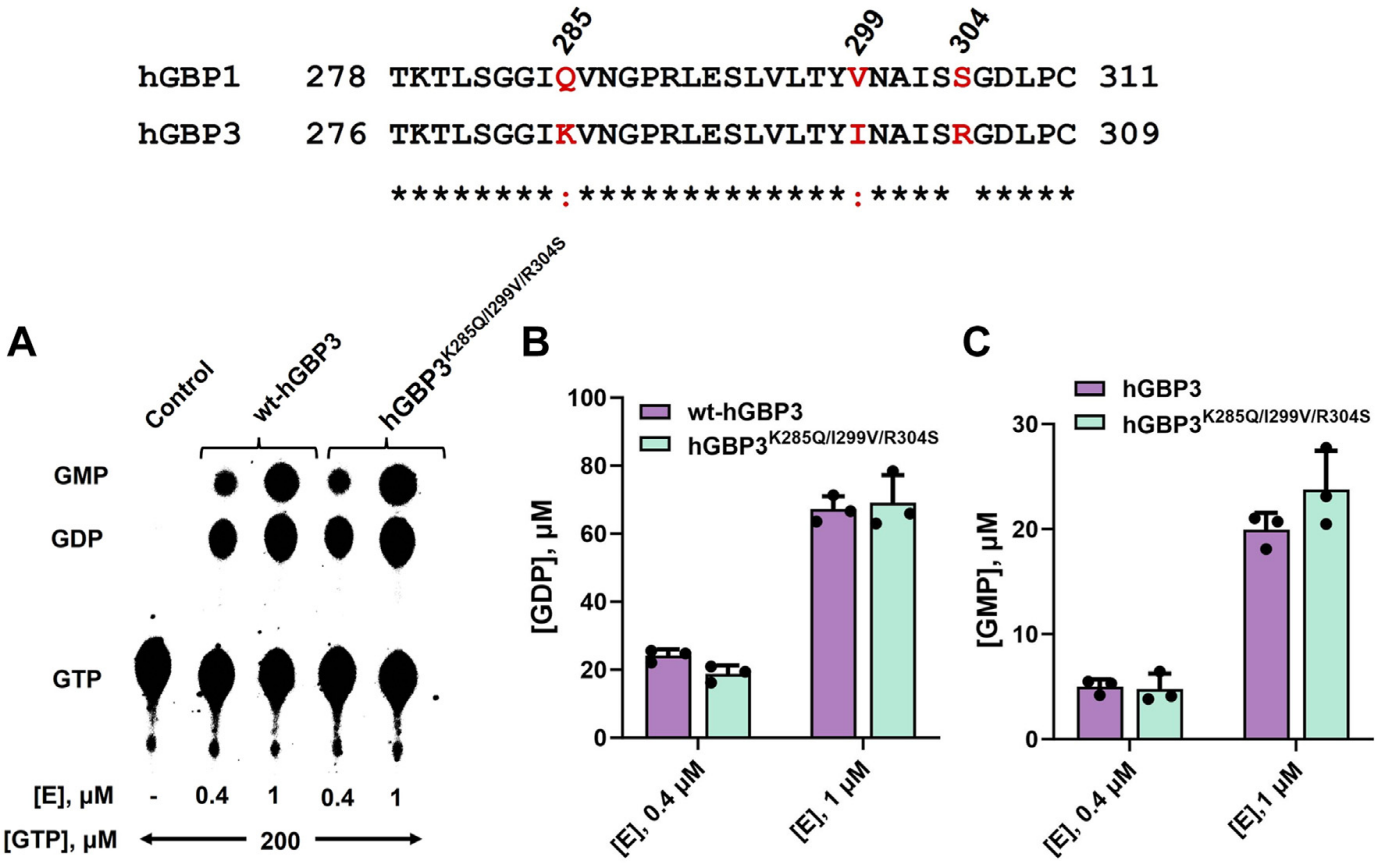
**Effect of intermediate region variation on GTPase activity.***A*, GTPase assay of wt-hGBP3 and hGBP3^K285Q/I299V/R304S^ at 0.4 and 1 μM protein concentrations. Bar graph representation of GDP (*B*) and GMP formation (*C*), respectively, estimated from the activity assays. These values are presented as averages with SDs, based on three independent experiments. GBP, guanylate-binding protein.

Our data indicate that the catalytic domain of hGBP3 requires its intermediate region for GMP formation. Sequence analysis of the intermediate region between hGBP3 and hGBP1 reveals that hGBP3 contains three residue variations. To check whether these variations have any effect on the GMP formation, we prepared a triple mutant hGBP3^K285Q/I299V/R304S^ in which K285, I299, and R304 of the intermediate region in hGBP3 were substituted with their corresponding residues of hGBP1. The overall product formation in this mutant was comparable to that of the wt-hGBP3 (Fig. 5, *A* and *C*), suggesting that the sequence variations of the intermediate region of hGBP3 do not affect the product formations, including GMP.

We recorded the CD spectra of the two truncated proteins in the far-UV range to check the effect of domain deletion on the secondary structure. We also recorded the CD spectra of the full-length protein for comparison. The observation of negative bands in full-length hGBP3 at 208 and 222 nm is indicative of a typical alpha-helical protein. The spectral patterns of the truncated variants were similar to those of the full-length protein (Fig. 4*E*). However, the CD intensities were low, indicating that the decrease in CD values in these truncated proteins is primarily due to the absence of a helical domain. The data also suggest that the deletion of the helical domain alone or its combination with the intermediate region does not have an impact on the overall secondary structure of these variants. We also recorded the CD spectra of the aforesaid triple mutant, which displayed a secondary structure similar to that of the WT protein (Fig. S4).

Overall, these results suggest that the helical domain of hGBP3 does not promote GMP formation, whereas the intermediate region along with the catalytic domain is essential for GMP production. The inability of the helical domain to promote GMP formation may be due to alteration in the helical domain–mediated interdomain interactions (see the Discussion).

### Residue variation in and around the catalytic site has no impact on GTP hydrolysis

**Figure 6:**
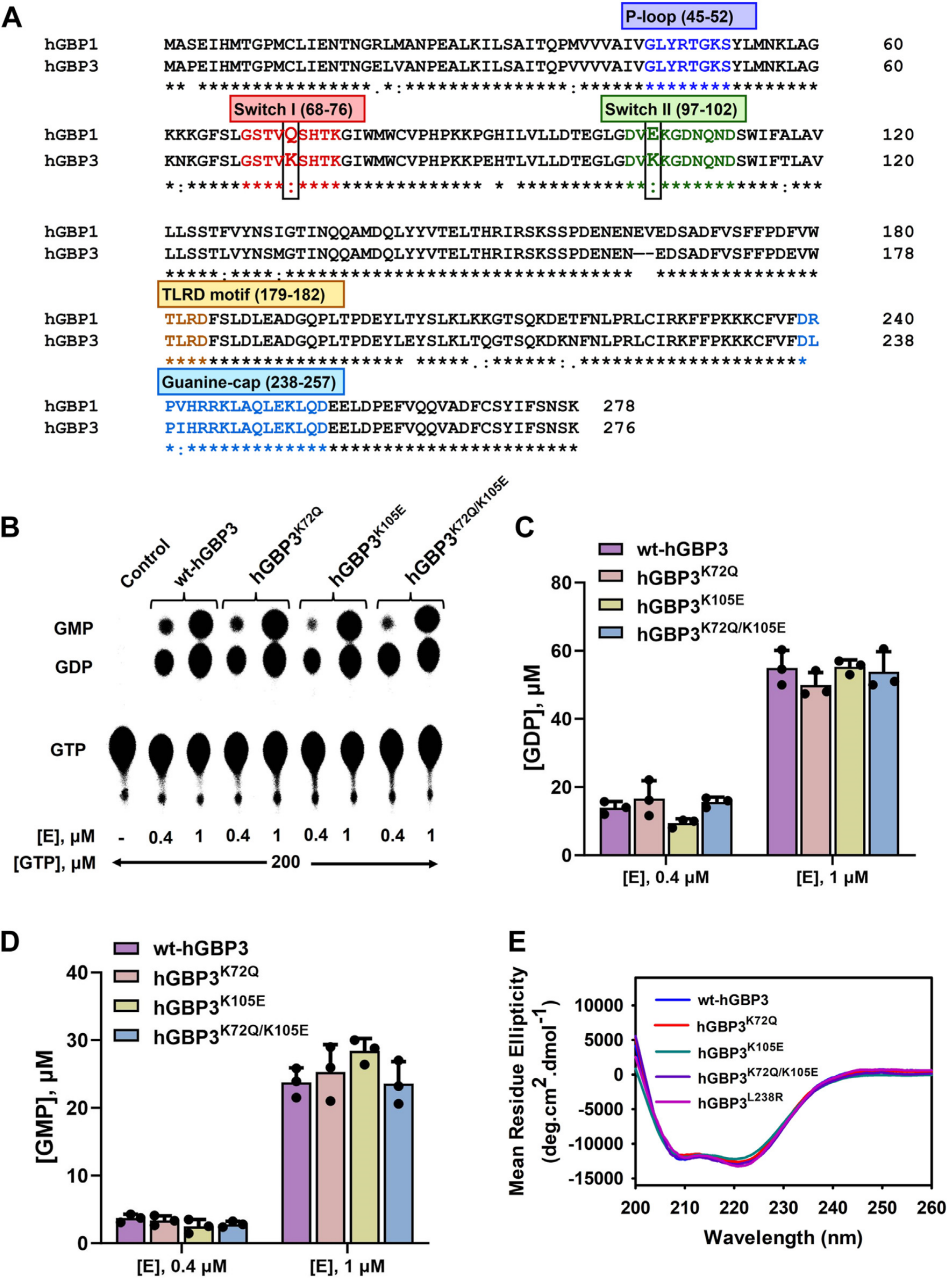
**Effect of switch I and switch II residues variation on GTP hydrolysis.***A*, a comparison of the sequences in the catalytic domain between hGBP1 and hGBP3. The catalytically significant regions are labeled in *boxes*. Variations in residues within the switch I and II regions are highlighted within a *black* box. *B*, GTPase assay of hGBP3 and its switch region mutations at protein concentrations of 0.4 and 1 μM. The figure shown here represents the outcome of one of the activity assay experiments. *C* and *D*, the data generated from these assays was used to calculate the production of GDP (*C*) and GMP (*D*) as presented in bar graphs. The data shown here in these two graphs is the average ± SDs of three independent experiments. It should be noted that under the experimental conditions, all mutants were in complex with GTP, as estimated by their *K*_d_ values. *E*, circular dichroism measurements were conducted on wt-hGBP3 and switch region mutants. The data displayed here are the averages of three independent measurements. GBP, guanylate-binding protein.

Thus far, our data suggest that neither the helical domain nor the intermediate region residue variations affect GMP production. Thus, we examined the regions in and around the active site of the catalytic domain. The active site of hGBPs consists of a substrate-binding pocket, switch I (63–76), switch II (97–111), and a guanine cap (237–255) region. The switch I contains the catalytic machinery. In hGBP1, switch I and switch II regions exist as a loop, and they undergo major structural changes upon GTP binding and hydrolysis (21). These structural changes are vital for stimulating GTPase activity, ultimately leading to enhanced production of GMP. A sequence comparison of these two regions (switch I and switch II) between hGBP3 and hGBP1 displays a few residue variations (Fig. 6*A*). In switch I, hGBP3 has a Lys at position 72 instead of a Gln. In switch II, Lys is found at position 105 instead of Glu. These two residues of hGBP3 were individually substituted with the corresponding residues of hGBP1 (hGBP3^K72Q^ and hGBP3^K105E^) to determine the effect of these variations on GMP formation. Activity assays of these mutants were performed at two different protein concentrations with a fixed concentration of the substrate. The overall product formation, including GMP, of these mutants was comparable to that of WT protein (Fig. 6, *B*–*D*). These results suggest that the variation of residues in these two switch regions does not affect GMP formation. A double mutant was also prepared, in which the residues of both switch regions of hGBP3 were substituted with that of hGBP1 (hGBP3^K72Q/K105E^) to check whether the replacement of these two residues together can increase GMP formation. However, this mutant did not increase GMP formation but rather showed similar to the WT. GDP formation was also comparable. These observations suggest that neither of the switch regions' residues variation affects GMP production.

**Figure 7:**
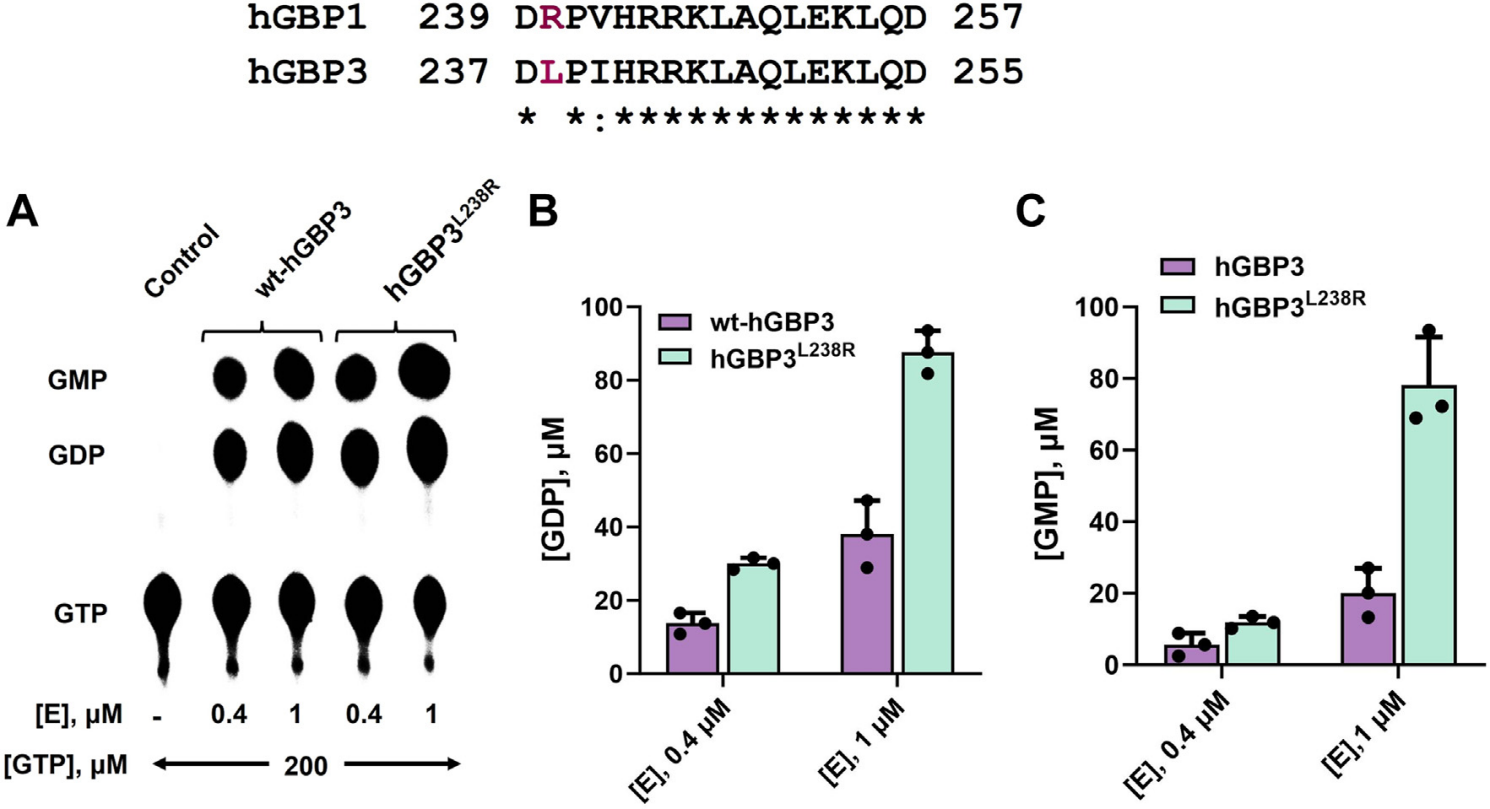
**Effect of guanine cap residue variation on GTPase activity.***A*, GTPase assay of wt-hGBP3 and hGBP3^L238R^ at protein concentrations of 0.4 and 1 μM. *B* and *C*, the bar graphs represent the average GDP and GMP production, respectively, based on the results obtained from the activity assays. Error bars in each figure correspond to the SDs of three independent experiments. GBP, guanylate-binding protein.

We also examined the sequence of the guanine cap (G-cap), which is located near the active site. In comparison to hGBP1, we noticed that the G-cap region of hGBP3 had two residues variation. However, one of them is major based on the property of a side chain (Fig. 7). hGBP3 has a Leu at position 238 instead of an Arg. It was earlier proposed that following the first phosphate cleavage of GTP in hGBP1, the G-cap movement plays a role in the repositioning of the nucleotide (26). This repositioning helps to bring the nucleotide toward the catalytic machinery for the occurrence of the second phosphate cleavage. Therefore, to check whether the above variation in the G-cap of hGBP3 has any effect on GMP formation, the hGBP3^L238R^ mutant was prepared. The activity assay at two different protein concentrations showed an increase in both product formations (Fig. 7, *A*–*C*). GMP was increased slightly more (∼2.5-fold increase in GMP *versus* ∼2-fold increase in GDP). However, compared to hGBP1, this amount of GMP is still considerably lower. This suggests that the residue variation in the G-cap region of hGBP3 has some effect on lower GMP formation, but it cannot fully account for the deficiency in enhanced GMP production.

We measured the mutant proteins’ substrate-binding affinity using N-methylantraniloyl (mant)-GppNHp, a fluorescently labeled substrate analog, to confirm that the mutant proteins are forming a complex with the substrate in the activity assay experiments. For comparison, we performed a similar experiment with the WT hGBP3. In this experiment, the concentration of the substrate was kept constant, but the protein concentration was increased. The fluorescence intensities change was plotted against the protein concentrations and fitted to a binding equation, which yielded a *K*_d_ value (Fig. S5). The *K*_d_ values for all the mutants were comparable with that of the WT hGBP3 (Table S1). Since the activity assays were conducted at a substrate concentration much higher than the substrate-binding affinity (under these experimental conditions, nearly 99% of the enzyme exists in the substrate-bound complex), a small variation in the *K*_d_ value is unlikely to influence the substrate hydrolysis.

We investigated the effect of residue mutations in switch regions (switch I and II) and the guanine cap on the protein’s secondary structure. For this purpose, the CD spectra of the mutants were recorded, and the results were compared to those of the WT protein. CD intensities of the mutant proteins were comparable to those of the WT (Fig. 6*E*), suggesting that these mutations in hGBP3 do not affect the overall secondary structure.

### Lack of proper catalytic loop repositioning leads to lower GMP formation

Our findings thus far indicate that the sequence differences in and around the active site are not fully responsible for the deficiency in enhanced GMP formation. It may be possible that hGBP3 and hGBP1 possess distinct tertiary structures in and around their active sites, preventing hGBP3 from producing more GMP. It has been shown that in hGBP1, the movement of the switch I loop containing the catalytic machinery is required to stimulate the second phosphate cleavage, leading to enhanced GMP production. This is necessary since it has been proposed that the same catalytic machinery be used for the cleavage of both phosphates of GTP. The repositioning of the catalytic loop requires two events: (i) the formation of an H-bond between the main chain carbonyl of K76 and the indole N of W79; and (ii) the movement of W79 toward the solvent (18, 25). Notably, W79 is situated in the β2 strand, which is located just after switch I. K76 is a part of the switch I loop. The combination of these two events facilitates bringing the catalytic machinery near the beta phosphate of GTP following the gamma phosphate cleavage so that the same catalytic machinery can be utilized for efficient second phosphate cleavage. Since W79 is conserved in this protein (Fig. 1), we sought to determine if there are deficiencies in the aforementioned two steps of hGBP3 that could explain the reduced GMP formation.

#### The W79 side chain is predicted to form an H-bond with the backbone carbonyl of K76 in the substrate-bound hGBP3

**Figure 8:**
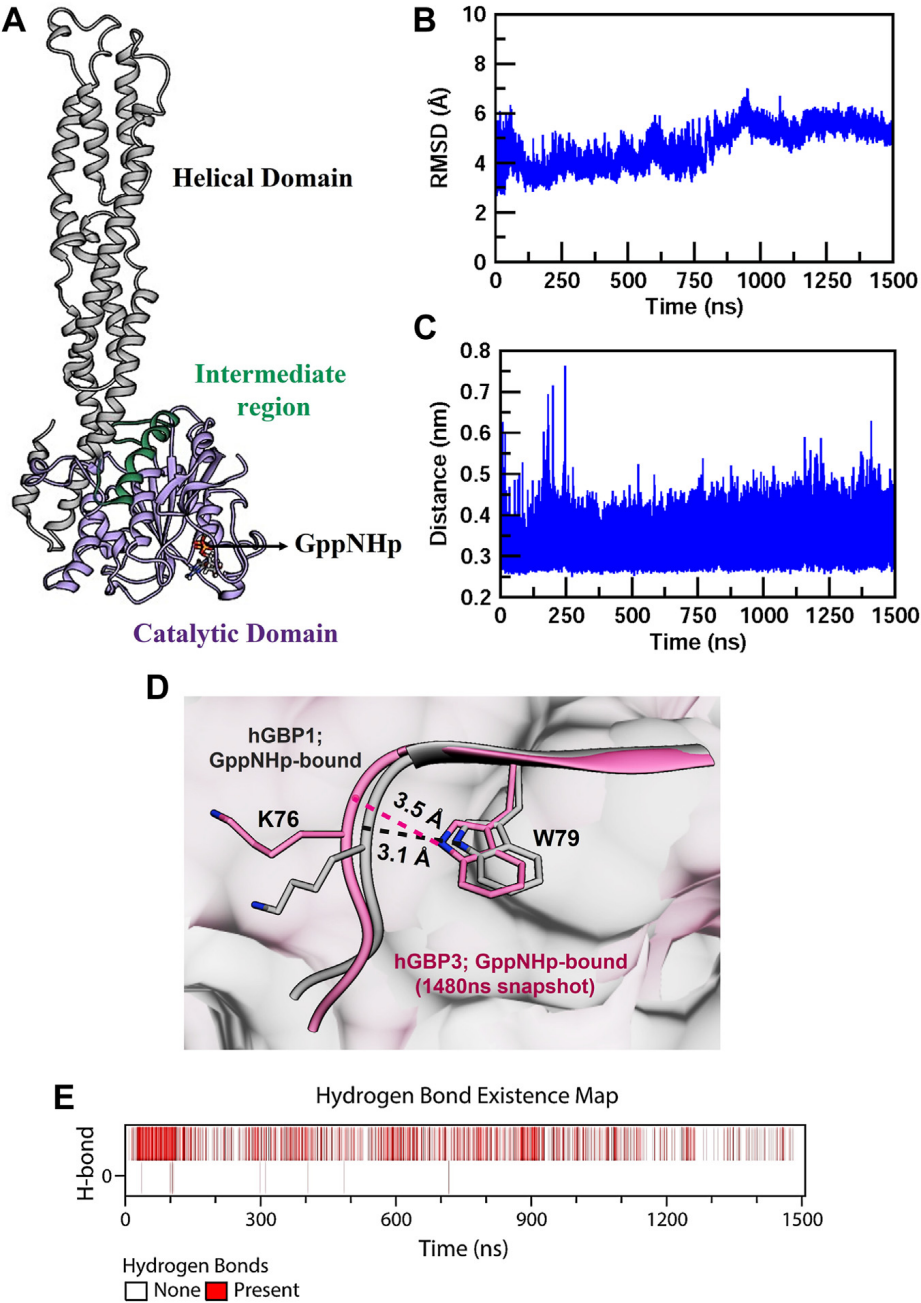
**Hydrogen bond between W79 indole N and K76 carbonyl O in the substrate-bound hGBP3.***A*, simulated structure of hGBP3 bound to GppNHp, representing an 800 ns snapshot, generated using UCSF Chimera software. It describes different domains within the structure. *B*, root mean square deviation (RMSD) of the protein backbone, calculated after least-squares fitting of the initial structure during a 1500 ns molecular dynamics simulation. *C*, graph representing the distance between the main-chain carbonyl O of K76 and the indole N of W79 in the GppNHp-bound hGBP3 structure, calculated for the time course of the simulation. *D*, an overlay between the crystal structure of GppNHp-bound hGBP1 (in *pink*, PDB: 1f5n) and the simulated structure of GppNHp-bound hGBP3 at the 1480 ns snapshot (in *purple*). This comparison illustrates the distance between the main-chain carbonyl O of K76 and the indole nitrogen of W79 using the UCSF Chimera software. *E*, a map illustrates the possible existence of hydrogen bonds during the simulations. GBP, guanylate-binding protein.

In hGBP1, the H-bond formation between the main chain CO of K76 and the side chain of W79 takes place only after the substrate binding (this contact is absent in the free protein). To check the presence of the above contact in the substrate-bound hGBP3, its 3D structure is required, which is not yet available. In the absence of this structure, we prepared a model structure of the substrate-bound hGBP3 using the crystal structure of hGBP1 bound to GppNHp (PDB: IF5N), since these two homologs show very high sequence identity (∼87%). The indole N of W79 makes an H-bonding contact with the main chain CO of K76 in the substrate-bound hGBP3 structure. To assess the stability of this contact, we conducted molecular dynamic simulation studies using the model structure for 1500 ns (Fig. 8*A*). The substrate-bound protein's backbone RMSD values showed equilibrium structures after 200 ns, which remained essentially constant till 800 ns. The RMSD values slightly increased after this time point and remained similar for the rest of the simulations (Fig. 8*B*). Analysis of the structures at different time points showed that the distance between the above two atoms varies from 3.4 to 4 Å. To check it further, we plotted this distance for the full simulations and found that it was around 3.5 Å after 250 ns (Fig. 8, *C* and *D*). This suggests the presence of a hydrogen bond between the W79 indole N and the K76 carbonyl O, which is consistent with the possibility of nearly 28% hydrogen bond formation (see the H-bond existence map, Fig. 8*E*).

**Figure 9:**
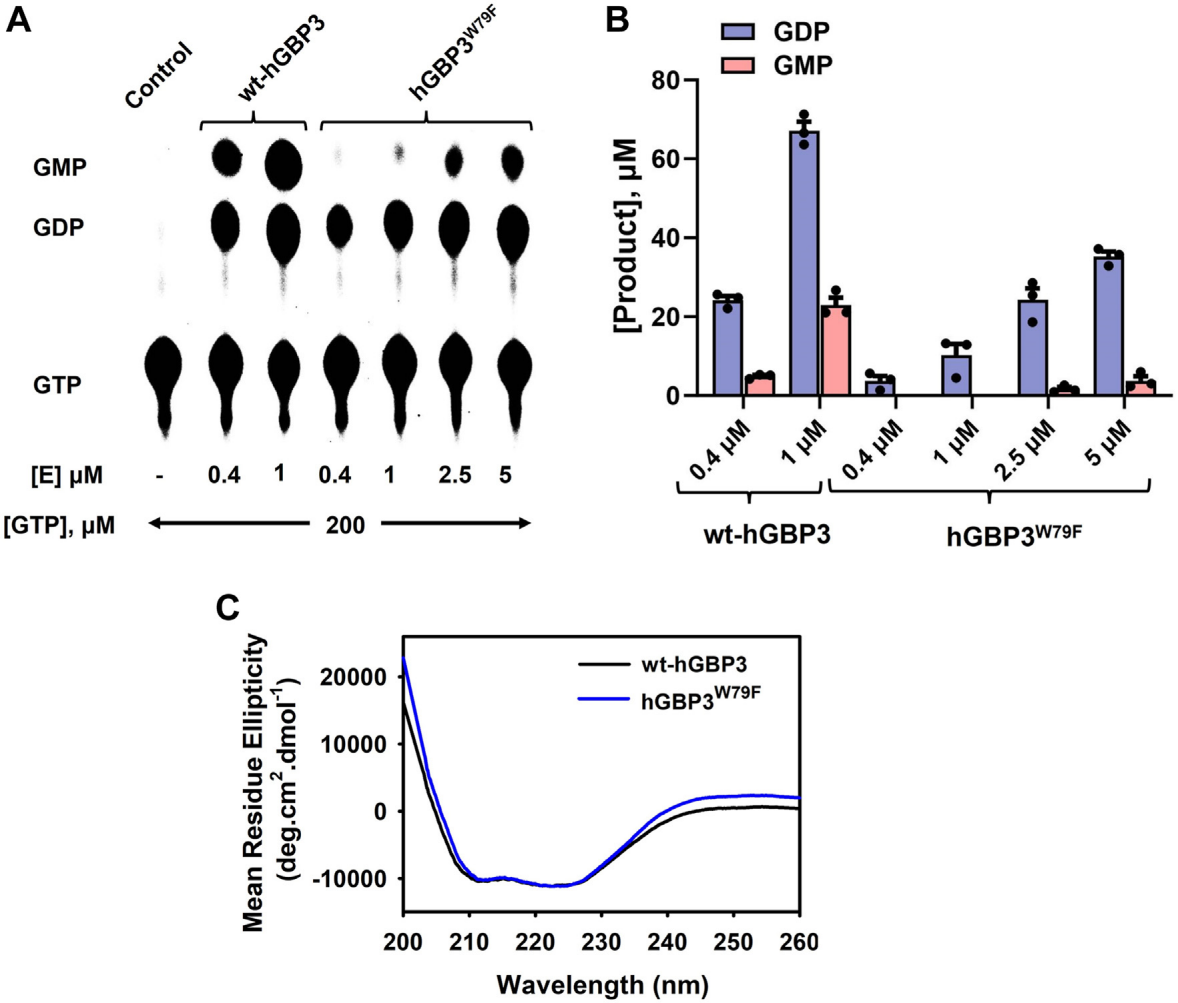
**The hydrogen bond between W79 indole N and K76 carbonyl O of hGBP3 plays a crucial role in GMP production.***A*, GTPase assay of wt-hGBP3 and hGBP3^W79F^ at varying concentrations of protein and 200 μM of GTP. *B*, the bar graph represents the average GDP and GMP production, based on the results obtained from the activity assays. The values are depicted as averages with SDs, based on three independent experiments. Under the experimental conditions, both proteins were in complex with the substrate, as confirmed by their *K*_d_ values. *C*, circular dichroism measurements of wt-hGBP3 and the hGBP3^W79F^ mutant. The graph presented here represents the averages of three independent measurements. GBP, guanylate-binding protein.

Next, we created the hGBP3^W79F^ mutant, where the above H-bond formation is disrupted but the hydrophobicity at position 79 is mostly preserved. We then performed its GTPase assay to understand the effect of the H-bond on GMP production, if this contact exists in the WT protein. Activity assay was performed at two concentrations of the mutant protein (0.4 and 1 μM) where the substrate concentration was kept at 200 μM. We also performed a similar assay with the wt-hGBP3 for comparison. Both products of this mutant were affected compared to those of the WT. However, GMP production was drastically reduced (Fig. 9, *A* and *B*). To check it further, we performed a similar assay with higher concentrations of the mutant protein (2.5 and 5 μM). This displayed GMP, which is significantly lower than that of the WT protein at 0.4 and 1 μM concentrations. These findings showed that the W79F mutation in hGBP3 considerably decreases GMP production, suggesting the importance of the H-bond in GMP formation. This also implies the existence of the aforementioned H-bond in the substrate-bound hGBP3, consistent with our molecular dynamics (MD) simulation studies. We believe that this bond remains intact during the first and second steps of GTP hydrolysis by hGBP3.

We also conducted the substrate-binding experiment with the hGBP3^W79F^ mutant to ensure that this protein formed a complex with the substrate during the activity assay experiment. The *K*_d_ value of this mutant was approximately 2.5-fold higher than that of the WT (Fig. S6). However, this variation in the *K*_d_ value is unlikely to influence the substrate hydrolysis, as nearly 99% of the enzyme formed a complex with the substrate in the above experimental conditions. Furthermore, we performed a CD measurement of this mutant which showed a spectrum similar to the WT protein (Fig. 9*C*), indicating that the W79F mutation in hGBP3 does not affect the secondary structure.

#### W79 of hGBP3 does not undergo solvent-exposure following the first phosphate cleavage of GTP

**Figure 10:**
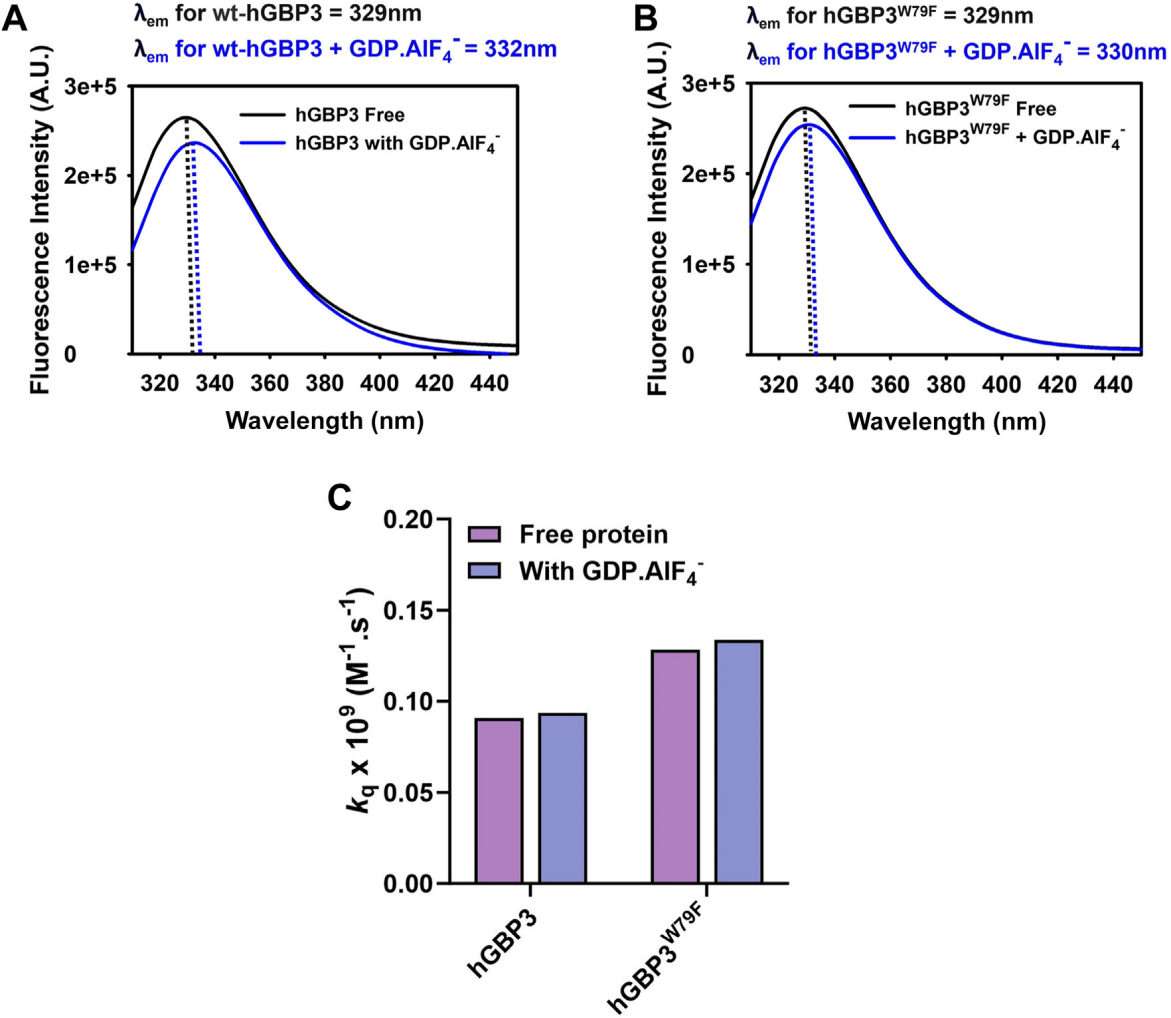
**W79 does not undergo a conformational change in hGBP3.** Tryptophan fluorescence spectra of (*A*) wt-hGBP3 and (*B*) the hGBP3^W79F^ mutant. Fluorescence scans of both proteins were recorded in the presence and absence of the GDP.AlF_4_^−^, transition state analog. The dotted vertical lines on the spectra indicate the emission maximum points. *C*, the graph shows the bimolecular quenching constant, *k*_q_ value, for both wt-hGBP3 and its W79F mutant. This plot demonstrates that the solvent accessibility of tryptophans in both proteins did not vary in the presence of GDP.AlF_4_^−^. *k*_q_ values are derived from the ratio of K_sv_ and τ_m_, where these values are estimated from separate experiments. Therefore, the *k*_q_ values are represented without the error bar. GBP, guanylate-binding protein.

Next, we sought to examine whether wt-hGBP3 undergoes a conformational change resulting in increased solvent accessibility of tryptophans following the gamma phosphate cleavage of GTP. If so, whether it is mediated by W79. Thus, we first recorded the intrinsic tryptophan fluorescence of WT hGBP3 both in the absence and presence of GDP.AlF_4_^−^, a well-known transition state analog for the first phosphate cleavage step of GTP. According to studies on several GTPases, this complex resembles the gamma phosphate hydrolysis step of GTP (GDP.P_i_), in which AlF_4_^−^ displays a configuration similar to P_i_. The fluorescence emission maximum of wt-hGBP3 with GDP.AlF_4_^−^ showed a red shift of approximately 2 nm, with a slight decrease in the fluorescence intensity (Fig. 10*A*), suggesting that some of the tryptophan(s) might be slightly exposed toward the solvent after the first phosphate cleavage step. Although this change in the emission maximum seems to be marginal, we wanted to check if it is mediated by W79. Therefore, we performed similar fluorescence studies with the hGBP3^W79F^ mutant. This variant did not nearly exhibit a change in the emission spectrum with GDP.AlF_4_^−^ (Fig. 10*B*), indicating that the slight movement of tryptophan toward the solvent in the WT protein appears to be mediated by W79.

To check this, we determined the *k*_q_ value, which is an accurate measure of the solvent accessibility of tryptophans. This was done by combining steady-state and time-resolved fluorescence studies with the WT or its W79F mutant with and without GDP.AlF_4_^−^. We performed dynamic fluorescence quenching of the protein tryptophans using CsCl as a well-established quencher [Q]. The Stern-Volmer quenching constant (K_sv_) was estimated by plotting F_0_/F against [Q], where F_0_ and F represent the fluorescence intensities without and with the quencher, respectively (Fig. S7). To calculate the *k*_q_ value, we also determined the τ_m,_ which is related to K_sv_/*k*_q._ This was carried out using time-resolved fluorescence decay kinetics of the protein. The τ_m_ value was obtained by fitting the decay kinetics to a sum of three exponential functions. The values for K_sv_ and τ_m_ are listed in Table 1. The *k*_q_ value of the wt-hGBP3 in the free form was similar to that of GDP.AlF_4_^−^ (Fig. 10*C*). This observation indicates that the tryptophans in the WT protein do not become solvent-exposed following the first phosphate cleavage. The *k*_q_ value of the W79F mutant also remained similar in the presence of GDP.AlF_4_^−^. Thus, unlike in hGBP1, W79 of hGBP3 does not undergo solvent exposure after the first phosphate cleavage step of GTP. This could be due to the lack of the helical domain–mediated specific interactions with the catalytic domain.

**Table 1:** Time-resolved fluorescence decay parameters obtained from the analysis using a sum of the three-exponential function^a^

Protein	α_1_	*τ*_1_ (ns)	α_2_	*τ*_2_ (ns)	α_3_	*τ*_3_ (ns)	*τ*_m_ (ns)	K_sv_ (M^−1^)	*k*_q_ × 10^−9^ (M^−1^ × s^−1^)
hGBP3	0.33	0.29	0.42	2.23	0.25	5.91	2.6 ± 0.05	0.22 ± 0.01	0.09
hGBP3 + GDP.AlF_4_^−^	0.36	0.21	0.23	5.5	0.4	2.11	2.2 ± 0.2	0.19 ± 0.016	0.089
hGBP3^W79F^	0.22	1.36	0.45	3.45	0.32	6.74	4.04 ± 0.3	0.48 ± 0.06	0.128
hGBP3^W79F^ + GDP.AlF_4_^−^	0.21	1.03	0.47	3.27	0.32	6.67	3.8 ± 0.3	0.46 ± 0.03	0.132

a The mean fluorescence lifetime was computed using the equation, τ_m_ = ∑α_i_ × τ_*i*_, *i* = 1 to 3. The α_i_ and τ_*i*_ values shown here correspond to one measurement. However, *τ*_m_ values represent the average of three independent experiments with SEM. K_sv_ was determined from the same number of measurements. The bimolecular quenching constant, *k*_q_, was calculated from the ratio of K_sv_ and *τ*_m_. Hence, the errors associated with its values are not shown.

**Figure 11:**
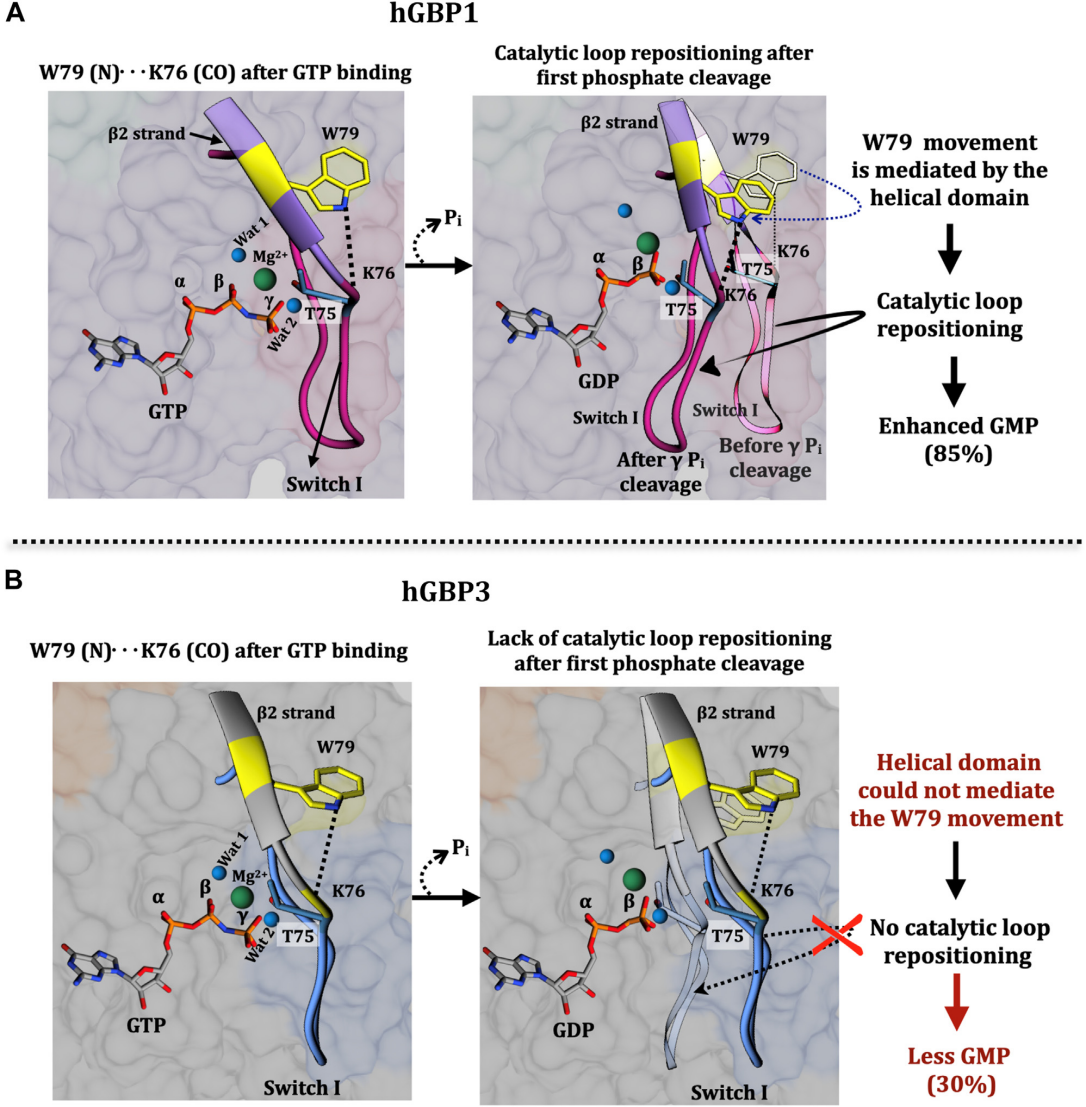
**Schematic representation of substrate binding and hydrolysis in hGBP1 and hGBP3.***A*, in the *left panel*, the active site of the substrate-bound hGBP1 has been shown based on the crystal structure (PBD: 1F5N). The positioning of the catalytic residue, T75, is highlighted in a *white background*. After GTP binding in hGBP1 W79 (located in the β2 strand), indole N makes an H-bonding contact with the K76 carbonyl of switch I loop. The *right panel* describes a schematic representation of the next step. Following the first phosphate cleavage of GTP, the helical domain mediates the movement of W79 toward the solvent, which in turn helps in the catalytic loop repositioning, so that T75 can be present at an optimal distance to carry out the second phosphate cleavage efficiently, leading to enhanced GMP formation. *B*, in the simulated structure of GppNHp-bound hGBP3, the indole N of W79 forms an H-bond with the backbone carbonyl of K76 (*left panel*). However, the helical domain fails to mediate the W79 movement toward the solvent after the first phosphate cleavage step. This results in the absence of catalytic loop repositioning toward the beta phosphate, which ultimately leads to the lower GMP formation (*right panel*). T75 of hGBP1 is conserved in hGBP3 and hence can be considered as a catalytic residue in hGBP3. GBP, guanylate-binding protein.

These findings imply that, despite the H-bond being formed between the side chain of W79 and the main chain carbonyl of K76 in the substrate-bound hGBP3, W79 does not become solvent-exposed following the first phosphate cleavage. This prevents the catalytic loop from being repositioned, which appears to be the primary cause of the lower GMP formation (Fig. 11).

## Discussion

In the present study, we showed that despite sharing the high sequence identity with hGBP1 (∼87%), hGBP3 displays a considerably lower amount of GMP compared to hGBP1. Based on the truncated protein data, we suggest that specific interdomain interactions mediated by the helical domain of hGBP3 does not take place. Because of this, W79 present in the catalytic domain does not move toward the solvent, preventing the catalytic loop from being repositioned. These imply that the loss of proper catalytic loop repositioning following the first phosphate cleavage step is caused by the variation in the helical domain. This could be the primary cause of hGBP3's reduced GMP formation when compared to hGBP1.

One may ask, how do the variations in the helical domain alter the interdomain interactions in hGBP3? In hGBP1, R227 and K228 belonging to helix 4 of the catalytic domain interact with E556 and E563 of helix 12 and E575 of helix 13 of the helical domain, respectively (Fig. S8*B*) (32). It was suggested that the interdomain interactions mediated by the above residues in hGBP1 play an important role in stimulating GTPase activity, resulting in enhanced GMP production. These residues of the catalytic and helical domains are conserved in hGBP3, and the corresponding residues are R225, K226, E561, E554, and E573. A comparison of the simulated structure of the substrate-bound hGBP3 with that of the crystal structure of hGBP1 (PDB: IF5N) revealed that K226 of hGBP3 does not interact with E573. However, R225 is involved in electrostatic interactions with E554 and E561 (Fig. S8*A*). It may be noted that E561 of hGBP3 is present in a loop, in contrast to its corresponding residue E563 of hGBP1, which is located in helix 12. This indicates that E561 may not form strong contact with R225. The analysis also revealed that the orientation and arrangement of helices 12 and 13 present in the helical domain of hGBP3 are varied compared with those of hGBP1. The orientation and arrangement of other helices, helices 7 to 11, are also varied (Fig. S9). Because of these variations, it is possible that the residues belonging to the helical domain cannot make proper contact with the residues of the catalytic domain in hGBP3, thereby resulting in the alteration of specific interdomain interactions. Although simulation studies of hGBP3 offer some insights into the possible differences in the interdomain interactions, studies over longer periods may yield additional information on the dynamics of the interdomain interactions.

hGBP2 is a close homolog of hGBP1 and hGBP3, with nearly 78 and 77% sequence identity, respectively. Unlike hGBP1 and hGBP3, which produce GMP that is 85 and 30% of the total product, respectively, hGBP2 displays a significantly lower amount of GMP (∼5% of the total product). Recently, it has been reported that the large decrease in GMP production in hGBP2 is mainly because of a defect in the catalytic loop movement following the first phosphate cleavage step of GTP (25). Our present study suggests that hGBP3 is also deficient in the catalytic loop movement. Thus, one would wonder why hGBP3 produces more GMP than hGBP2 (30% *versus* 5% GMP), even though both proteins lack the catalytic loop movement. It should be noted that the two events—first, the formation of the H-bond between the side chain of W79 and the main chain carbonyl of K76 and second, W79's movement into the solvent—are both necessary for the above catalytic loop movement. These two events together bring the loop containing the catalytic residue toward the beta phosphate, resulting in efficient GMP formation. Our *in silico* study on wt-hGBP3 and the activity assay of its W79F mutant together suggest the presence of the aforesaid H-bond in the substrate-bound hGBP3. The disruption of this interaction in the W79F mutant of hGBP3 led to a significant decrease in GMP formation (∼5% of the total product), which is comparable to the GMP produced by WT hGBP2. We also observed that W79 of hGBP3 does not move toward the solvent after the first phosphate cleavage of GTP. The above H-bond is absent in hGBP2, but W79 is exposed to the solvent (25). These findings imply that the existence of the aforementioned H-bond accounts for the 25% (5%–30%) higher GMP production in hGBP3 than hGBP2. This also suggests that only the formation of the H-bond in these proteins can somewhat help the cleavage of the second phosphate, which appears to be due to a slight movement of the catalytic loop toward the beta phosphate. It should be noted that the better repositioning of the catalytic loop, where W79 is exposed to the solvent coupled with the formation of the aforementioned H-bond, leads to a further increase in the GMP formation in hGBP1.

Furthermore, it may be suggested that, for stimulated GMP formation to occur in hGBP homologs, the above H-bond formation should take place first, followed by the conformational change of the protein that exposes W79 to the solvent. This also implies that unless the aforementioned H-bond is present, W79's solvent exposure does not affect the second phosphate cleavage.

Since the helical domain has been suggested to mediate the solvent exposure of W79 present in the catalytic domain (18), one may like to know, whether it can also influence the formation of the above H-bond. It may be noted that the GMP formation observed in the truncated hGBP3^309^ (lacking the helical domain) is comparable to that of the full-length protein. The aforementioned H-bond is found to exist in the substrate-bound full-length hGBP3. The observation of a similar amount of GMP in the full-length and truncated hGBP3 indicates that the H-bond is likely to be present in the substrate-bound hGBP^309^. This also implies that the helical domain does not seem to influence the formation of the H-bond. To verify the presence of the H-bond in the truncated protein, we created the W79F mutant in hGBP3^309^ and investigated how this mutation affected GMP formation. GMP production was significantly decreased, which is similar to that of the full-length hGBP3^W79F^ variant (Fig. S10). This finding suggests the presence of the above H-bond in the truncated protein and that has a role in GMP formation. Thus, it can be suggested that the helical domain of hGBP3 does not influence the H-bond formation between the main chain CO of K76 and the side chain of W79 in the substrate-bound protein.

WT hGBP3 produces GMP, which is 30% of the total product, whereas its G-cap mutant, L238R yields 40% GMP. This suggests that the single residue replacement in the guanine cap of hGBP3 with that of hGBP1 (hGBP3^L238R^) increases GMP production to some extent. One may ask, why does this mutation result in a 10% increase in GMP? As previously discussed, following the first phosphate cleavage of GTP in hGBP1, the G-cap plays a role in the nucleotide movement toward the catalytic machinery, enabling the second phosphate cleavage to occur. The better nucleotide repositioning is therefore likely to be the cause of the increased GMP production in the hGBP3^L238R^ mutant. This is supported by the double mutant, hGBP3^W79F/L238R^ (in which the above H-bond formation was hampered but the G-cap defect was primarily restored), which yielded GMP that is ∼12% of the total product (Fig. S11). This is slightly higher than the GMP observed in the hGBP3^W79F^ mutant (∼5%). The data also imply that the improper nucleotide movement following the first phosphate cleavage of GTP is partly responsible for lower GMP production in hGBP3 as compared to hGBP1.

The helical domain is known to mediate enhanced GMP formation in hGBP1 through stimulated GTPase activity. The same domain of hGBP3, however, is not involved in GTP hydrolysis. Thus, it is plausible to hypothesize that this domain is important for the protein’s biological function. It has been reported earlier that the hydrolysis of GTP by hGBP1 not only results in enhanced GMP production but also leads to the formation of substrate hydrolysis–induced protein assembly (24). hGBP3 may form a substrate hydrolysis–induced protein assembly, which could be required for its biological function(s). However, additional investigations are needed to validate this hypothesis.

In summary, we elucidated the role of the individual domains of hGBP3 in GTP hydrolysis. The catalytic domain alone can hydrolyze the gamma phosphate of GTP only, but in the presence of the intermediate region, it can further hydrolyze the beta phosphate, thereby showing the importance of the intermediate region in GMP formation. Contrary to hGBP1, hGBP3's helical domain plays no role in the regulation of GTP hydrolysis, leading to a lower GMP formation. This may be due to the differences in the structural arrangement of the helical domain, which might have prevented the helical domain from making specific contact with the catalytic domain, thereby preventing W79 from being exposed to solvents. Hence, after the first phosphate cleavage of GTP, the loop containing the catalytic residue cannot be redirected toward the beta phosphate, despite the formation of the prerequisite H-bond of W79 (Fig. 11). This is an essential step for efficient GMP formation since hGBP1 and hGBP2 utilize the same catalytic residue for both steps of the phosphate cleavages. Thus, this work provides an overview of the function of each hGBP3 domain in the hydrolysis of GTP and presents a possible molecular basis for the lower GMP formation by this protein. The study also offers insight that despite the conservation of the key regulatory residue, the hGBP homologs show variation in the product ratio, which may be associated with the difference in their antiviral activity.

## Experimental procedures

### Preparation of truncated hGBP3 variants

The truncated variants of hGBP3 were created through standard PCR amplification of hGBP3, using the WT hGBP3 as a template. This was done using a suitable reverse and forward primer, as shown in Table S2. The amplified genes were then cloned into the pET22b(+) vector with the help of the NdeI and XhoI restriction sites. Positive clones were verified *via* Sanger sequencing. The full-length hGBP1 and its truncated variant hGBP1^311^ constructs were used from a previous report (31).

### Generation of mutant constructs

Mutant constructs (K72Q, W79F, K105E, K72Q/K105E, L238R, W79F/L238R, K285Q/I299V/R304S) were prepared by site-directed mutagenesis technique using suitable reverse and forward primers, listed in Table S2. PCR amplification was performed with the help of HF Phusion polymerase from Pfu and Thermo Fisher Scientific, by following the procedure recommended by the manufacturer. Positive mutations were identified by DNA sequencing.

### Overexpression and purification of proteins

The *Escherichia coli* BL21(DE3) cells were individually transformed with plasmids that contained hGBP1, WT hGBP3, and various hybrid, truncated, and mutant gene variants. The hGBP1 protein was overexpressed and purified as previously reported (31). The expression temperatures for the WT hGBP3 and its protein variants were set at 25 °C. The induction of cells was carried out using 0.3 mM IPTG when the absorbance was 0.6 at 600 nm. Following this, the harvested cells were reconstituted in a buffer that included 50 mM Tris at pH 8.0, 30 mM imidazole, 500 mM NaCl, 5 mM MgCl_2_, 10% glycerol, 10 mM β-mercaptoethanol, 0.1% NP-40, along with protease inhibitor cocktail tablets. The cell lysis involved treating them with 0.5 mM lysozyme for 15 min, followed by sonication. The supernatant was obtained after centrifuging the cell lysate at the speed of 13,000 rpm for 45 min at 4 °C, then incubating it with Ni-NTA agarose beads (Qiagen). Proteins were extracted with 250 mM imidazole, and any proteins deemed contaminants were eliminated by performing size-exclusion chromatography with an appropriate column (Superdex 75 or 200). Purified proteins were then concentrated utilizing Amicon filters that had a size cutoff of 30 or 10 kDa. The concentrated proteins were flash-frozen and stored with 2.5 mM DTT and 10% glycerol at −80 °C. Lastly, Bradford reagent (BioRad) was used to determine the protein concentration.

The overexpression and purification of full-length hGBP1 and hGBP1^311^ were carried out using the reported procedure (31).

### GTPase assays

GTPase activity assays of WT hGBP3, truncated, mutant variants were performed using reaction buffer having tris (Sigma) 50 mM (pH 7.5), MgCl_2_ (Merck) 5 mM, KCl (Merck) 100 mM, DTT (Sigma) 0.2 mM, and a minute quantity of [α-^32^P] radiolabeled GTP (PerkinElmer). The enzyme and unlabeled GTP concentrations were varied depending on the experiment. The reaction was performed for 30 min at 37 °C and stopped by the addition of 125 mM EDTA (the final concentration). The reaction mixture was then subjected to thin-layer chromatography to separate the products. Polyethyleneimine cellulose sheets with 0.75 M KH_2_PO_4_, pH 3.5 buffer were used for this purpose. After separation, the polyethyleneimine cellulose sheets were exposed to a phosphor imaging plate for 12 to 16 h and then quantified using phosphorimagers (FLA-5100, Fujifilm and Amersham Typhoon, GE). The intensities and contrasts of the blots have been adjusted to generate presentable images.

### CD measurements

CD spectra of WT hGBP3, truncated, and mutant variants were recorded at wavelengths ranging from 200 to 260 nm (far-UV region) at 25 °C using a spectropolarimeter (ChiraScan, Applied Photophysics). A 1 mm quartz cuvette and a buffer having 20 mM Tris (pH 7.5), 5 mM MgCl_2_, and 100 mM KCl were used for this purpose. All the scans were recorded in triplicate, and their average was used for analysis. Buffer spectra were subtracted from the samples for baseline correction.

### Steady-state tryptophan fluorescence studies

Intrinsic tryptophan fluorescence measurements of WT hGBP3 and its W79F mutant were performed using a spectrofluorometer (Fluoromax 4, Horiba Scientific). Tryptophans were excited at 295 nm, and the emission spectra were recorded in the wavelength range 310 to 450 nm at room temperature. The monochromator slit width was maintained at 3 nm for both excitation and emission. These measurements were carried out using 1 μM of the protein and a reaction buffer comprising 50 mM Tris (pH 7.5), 5 mM MgCl_2_, and 100 mM KCl. To prepare the transition-state analog, GDP.AlF_4_^−^, we sequentially added 300 μM AlCl_3_ (Sigma), 10 mM NaF (Merck), and 200 μM GDP to the reaction buffer. Before spectrum acquisition, the proteins were incubated with the analog for 1 h at room temperature. Three independent experiments were conducted for each protein, and we applied inner filter effect correction to all fluorescence intensities in the steady-state spectra following the reported procedure (33).

Fluorescence quenching experiments on tryptophans were conducted with CsCl (Sigma) serving as the quenching agent, both in the presence and absence of the analog GDP.AlF_4_^−^. The proteins were subjected to titration with increasing CsCl concentrations, and at each incremental stage, excitation of tryptophans occurred at 295 nm, followed by the recording of emission spectra spanning from 310 to 450 nm. The quencher concentration was then plotted against F_0_/F, with F representing the fluorescence intensity in the presence of the quencher and F_0_ indicating the intensity in its absence. To analyze the data, the Stern-Volmer equation, F_0_/F = 1 + K_SV_ × [Q], was employed, where K_SV_ denotes the Stern-Volmer quenching constant and [Q] stands for the quencher concentration. Notably, K_SV_ is linked to *k*_q_ × τ_m_, with *k*_q_ representing the bimolecular quenching constant and τ_m_ representing the mean fluorescence lifetime.

### Time-resolved fluorescence

A time-correlated single-photon counting spectrophotometer (Edinburg FLS 920 model) was used to carry out time-resolved fluorescence measurements. Tryptophans in both WT and mutant hGBP3 variants were excited by a light-emitting diode emitting light at a central wavelength of 295 nm. Subsequently, the maximum emission was recorded using a single monochromator equipped with a 10 nm slit width, covering a period of 50 ns for proteins, in the presence and absence of GDP.AlF_4_^−^. The fluorescence decay profiles were typically acquired over 4095 channels, each providing a time resolution of 12.21 ps per channel until a count of 5000 was reached. For data analysis, the Decay Fit software was employed, incorporating the relevant instrument response functions obtained from scattering solutions. The collected instrument response function typically exhibited a full width at half maximum of approximately 130 ps. To evaluate the patterns of fluorescence intensity decay, a nonlinear iterative least-squares fitting approach was employed, utilizing the following equation: *I*(t) = Σα_i_exp(−t/τ_i_), where *I*(t) represents the fluorescence intensity at time t. The variable α_i_ corresponds to the amplitude associated with the fluorescence lifetime τ_i_, ensuring that the Σα_i_ = 1 (34).

### Measurement of substrate-binding affinity

The determination of protein substrate-binding affinities involved a titration process where a constant concentration of mant-GppNHp (obtained from Jena Bioscience) at 0.5 μM was incrementally titrated with protein. These measurements were conducted within a buffer solution comprising 50 mM Tris (pH 7.5), 5 mM MgCl_2_, and 100 mM KCl. During the experiments, mant-GppNHp was excited at a wavelength of 366 nm, and emission spectra spanning from 400 to 540 nm were recorded. Fluorescence intensity at 435 nm was plotted against protein concentration and fitted to the following quadratic equation to obtain the substrate binding affinity constants (*K*_d_) using Sigma Plot 12.5.

$$F=F_{\min}+\left(F_{\max}-F_{\min}\right)\frac{A_{0}+B_{0}+K_{d}-\sqrt{{\left(A_{0}+B_{0}+K_{d}\right)}^{2}-4A_{0}B_{0}}}{2B_{0}}$$

where A_0_ is the total protein concentration, B_0_ stands for the concentration of mant-GppNHp, F denotes the fluorescence intensity recorded at a specific protein concentration, F_min_ corresponds to the fluorescence intensity measured in the absence of protein, and F_max_ represents the highest fluorescence intensity recorded at the maximum protein concentration.

### MD simulations

The GppNHp-bound hGBP3 structure was generated with the help of the Swiss Model (35), using the crystal structure of GppNHp-bound hGBP1 (PDB ID: 1F5N) as a template. The model structure was validated by the Ramachandran plot, where majority of the residues were found in the most favored region (∼93%) (Fig. S12). The plot was generated using the PROCHECK server (36). MD simulations of the above model structure was conducted with the help of Gromacs 5.1.4 software (37). The GTP analog, GppNHp, was parameterized with a general AMBER force field (GAFF v1.5) using AM1-BCC charges utilizing AmberTools18. The topology parameters for hGBP3 were defined according to the standard AMBER99SB-iLDN forcefield format. Solvation of proteins was performed with explicit TIP3P water molecules within a triclinic simulation box, applying periodic boundary conditions. To maintain system neutrality, Na+ ions were added. After this, an energy minimization was carried out with the help of the steepest gradient minimization method. Equilibration of the system was performed in two stages: (i) 100 ps of NPT equilibrium (isothermal-isobaric ensemble) and (ii) 100 ps of NVT equilibrium (canonical ensemble). Position-restrained dynamics were employed using the LINC algorithm. During the simulations, the temperature was maintained at a constant 300 K, regulated using the Verlet algorithm where the coupling constant was 0.1 ps. Subsequently, a standard MD simulation was performed, with snapshots collected at every 10 ps. Key simulation parameters included the following: system designation as hGBP3-GppNHp, box dimensions of 7.8 × 9.2 × 15.50 nm³, 33,720 water molecules, 13 Na+ ions, and a simulation length of 1500 ns. The system pressure was controlled at 1 bar through the Parrinello-Rahman barostat where the coupling constant was 2 ps. A timestep of 2 fs was adopted for the integration of the equation of motion. The threshold distance for nonbonded interactions (Leonard-Johns and Coulomb) was kept at 1.4 nm. The electrostatic interactions were computed with the help of the particle mesh Ewald method where the grid spacing was set at 0.16 nm. The trajectory analysis was done by Gromacs utility programs. Finally, all the representative figures from simulation studies were created utilizing the UCSF Chimera software.

## Data availability

All data generated during this study are included in this article and its supporting information file.

## Supporting information

This article contains supporting information.

## Conflict of interest

The authors declare that they have no conflict of interest with the contents of this article.
