# Supplementary material for: Helical domain of hGBP3 cannot stimulate the second phosphate cleavage of GTP
Source: J Biol Chem. 2024 Jan 30;300(3):105696. doi: 10.1016/j.jbc.2024.105696 (PMC10910063; doi:10.1016/j.jbc.2024.105696)

**Supporting Information**

**Helical domain of hGBP3 cannot stimulate the second phosphate cleavage of GTP**

Divya Rashmi, Sowmiya Gupta, Tasneem Kausar, and Apurba Kumar Sau*

National Institute of Immunology, Aruna Asaf Ali Marg, New Delhi 110067, India

***** To whom correspondence should be addressed: Dr. Apurba Kumar Sau, National Institute of Immunology, Aruna Asaf Ali Marg, New Delhi, Delhi 110067, India; [apurba@nii.ac.in](file:///E:\..\AnkitaNme\AppData\Roaming\AppData\Local\AppData\Local\Temp\apurba@nii.res.in), [apurbaksau@gmail.com](file:///E:\..\AnkitaNme\AppData\Roaming\AppData\Local\AppData\Local\Temp\apurbaksau@gmail.com); Tel. +91-11-26703768; Fax. +91-11-26742125/26742626

**Table S1.***K*_d_ values determined from the substrate-binding affinity measurements of wt-hGBP3 and its mutant proteins. Error associated with the data represents standard error.

| **Proteins** | ***K*_d_ (μM)** |
| --- | --- |
| **Wt-hGBP3** | **2 ± 0.07** |
| **hGBP3^K285Q/I299V/R304S^** | **4.9 ± 1.6** |
| **hGBP3^K72Q^** | **2.1 ± 0.5** |
| **hGBP3^K105E^** | **1.4 ± 0.4** |
| **hGBP3^K72Q/K105E^** | **2 ± 0.2** |
| **hGBP3^L238R^** | **1.8± 0.1** |
| **hGBP3^W79F^** | **5.1 ± 0.5** |

**Table S2.**List of primers used for the preparation of wild type, truncated and mutant hGBP3.

| **Protein** | **Primer** | **Sequence (5’ to 3’)** |
| --- | --- | --- |
| **wt-hGBP3** | Forward | GCG CATATG ATG GCT CCA GAG |
|  | Reverse | GCG CTCGAG GAT CTT TAG CTT ATG |
| **hGBP3^309^** | Forward | GCG CATATG ATG GCT CCA GAG |
|  | Reverse | GCG CTCGAG GCA GGG CAG ATC CCC TCT |
| **hGBP3^276^** | Forward | GCG CATATG ATG GCT CCA GAG |
|  | Reverse | GCG CTCGAG TTT AGT TTT GGA |
| **hGBP3^K285Q^** | Forward | GGA GGC ATC CAG GTC AAT GGG |
|  | Reverse | CCC ATT GAC CTG GAT GCC TCC |
| **hGBP3^I299V^** | Forward | CTG ACC TAT GTC AAT GCT ATC |
|  | Reverse | GAT AGC ATT GAC ATA GGT CAG |
| **hGBP3^R304S^** | Forward | GCT ATC AGC AGT GGG GAT CTG |
|  | Reverse | CAG ATC CCC ACT GCT GAT AGC |
| **hGBP3^K72Q^** | Forward | TCC ACA GTG CAA TCT CAC ACC |
|  | Reverse | GGT GTG AGA TTG CAC TGT GGA |
|  |  |  |
| **hGBP3^K105E^** | Forward | CTG GGA GAT GTA GAG AAG GGT GACA |
|  | Reverse | TGTC ACC CTT CTC TAC ATC TCC CAG |
| **hGBP3^L238R^** | Forward | GTC TTC GAT CGG CCC ATT CAC |
|  | Reverse | GTG AAT GGG CCG ATC GAA GAC |
| **hGBP3^W79F^** | Forward | CT AAA GGA ATC TTC ATG TGG TGT GTG C |
|  | Reverse | G CAC ACA CCA CAT GAA GAT TCC TTT AG |


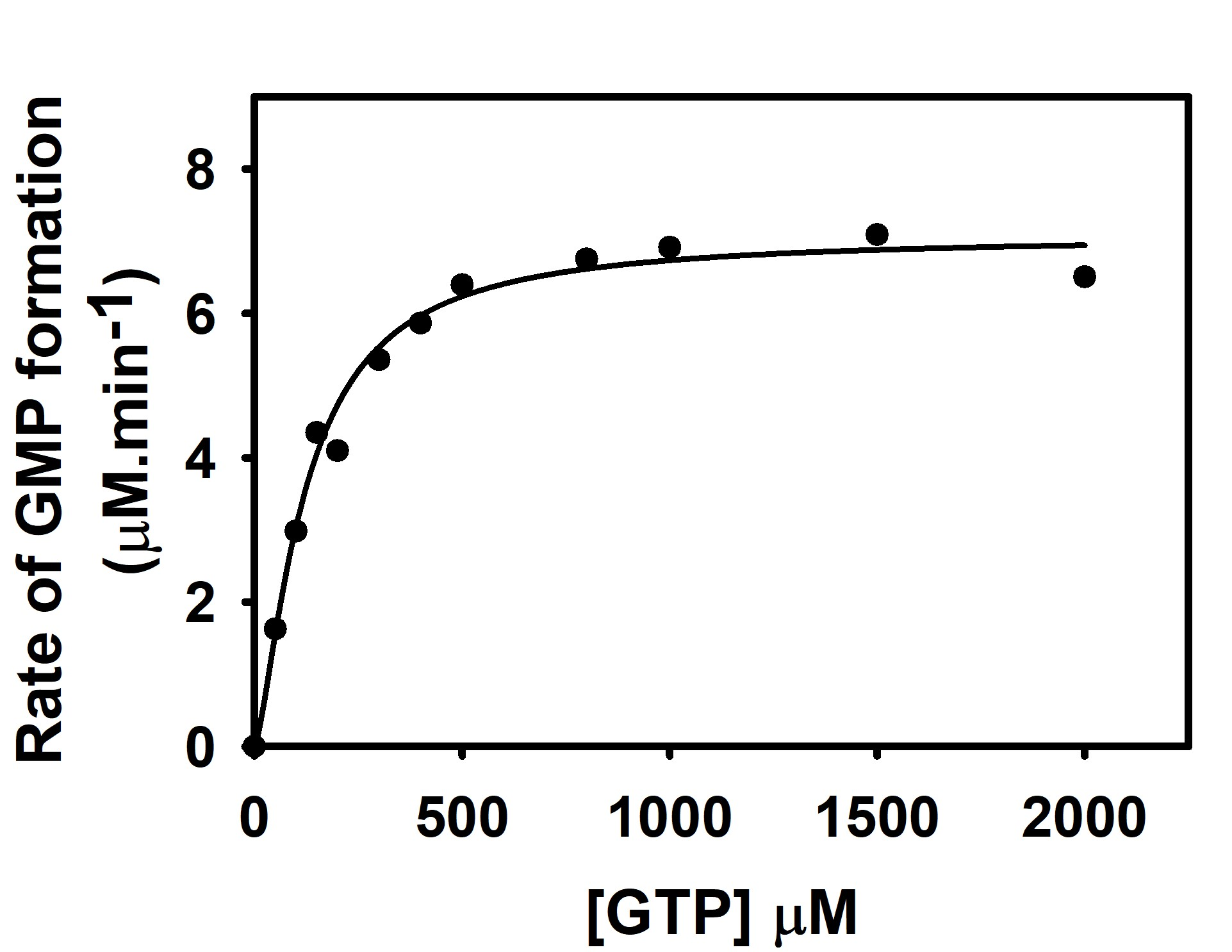

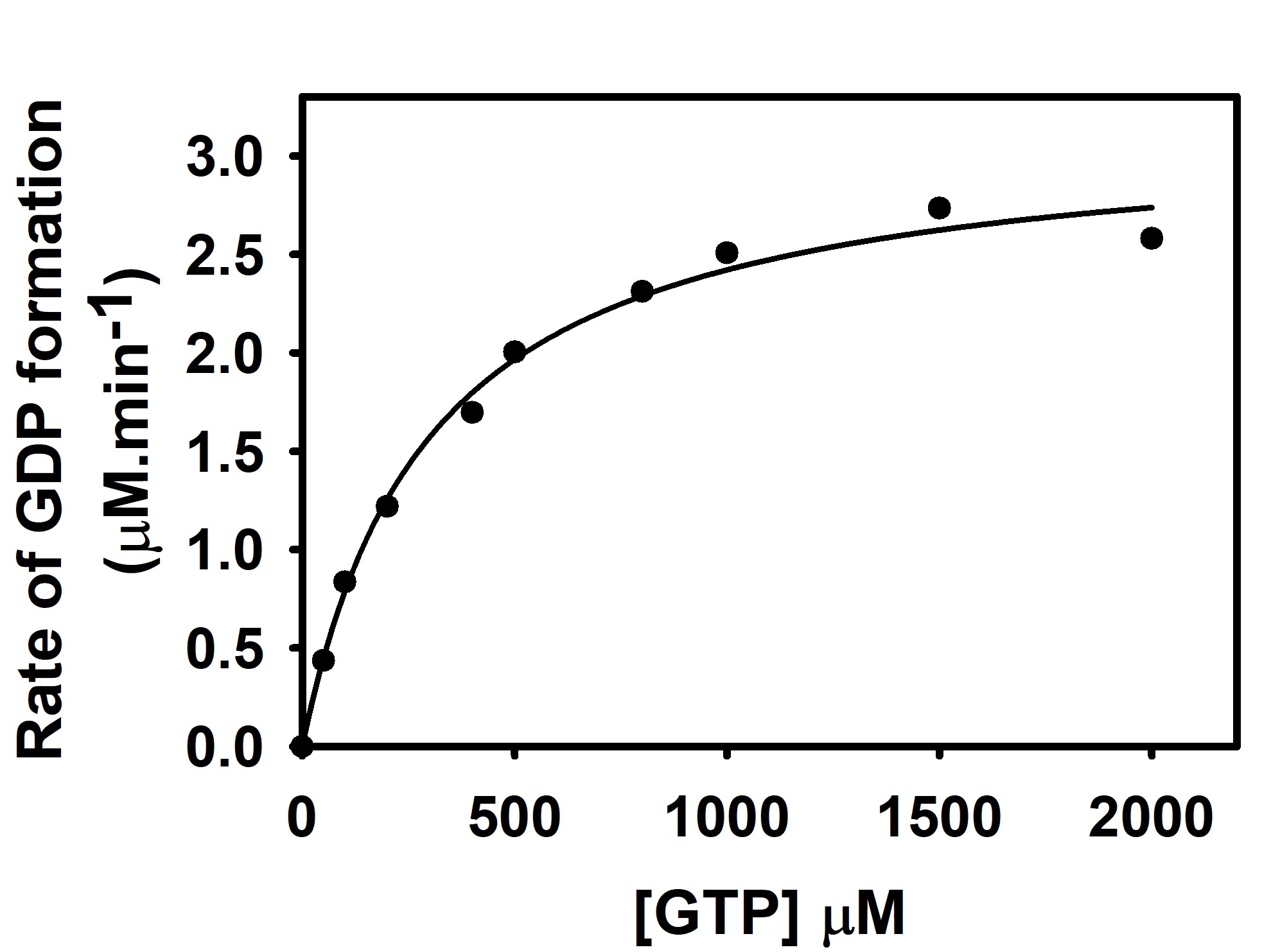


**Figure S1**. Steady-state kinetics of hGBP1. We used 0.4 μM protein and a minute quantity of radiolabeled [α-^32^ P] GTP but varied the unlabeled GTP concentration to 2mM as shown in the figure. The initial rates for each product were calculated and plotted against the concentration of GTP. The plot represents the rate of GDP (A) and GMP formation (B) versus GTP concentration.

**A**

**B**


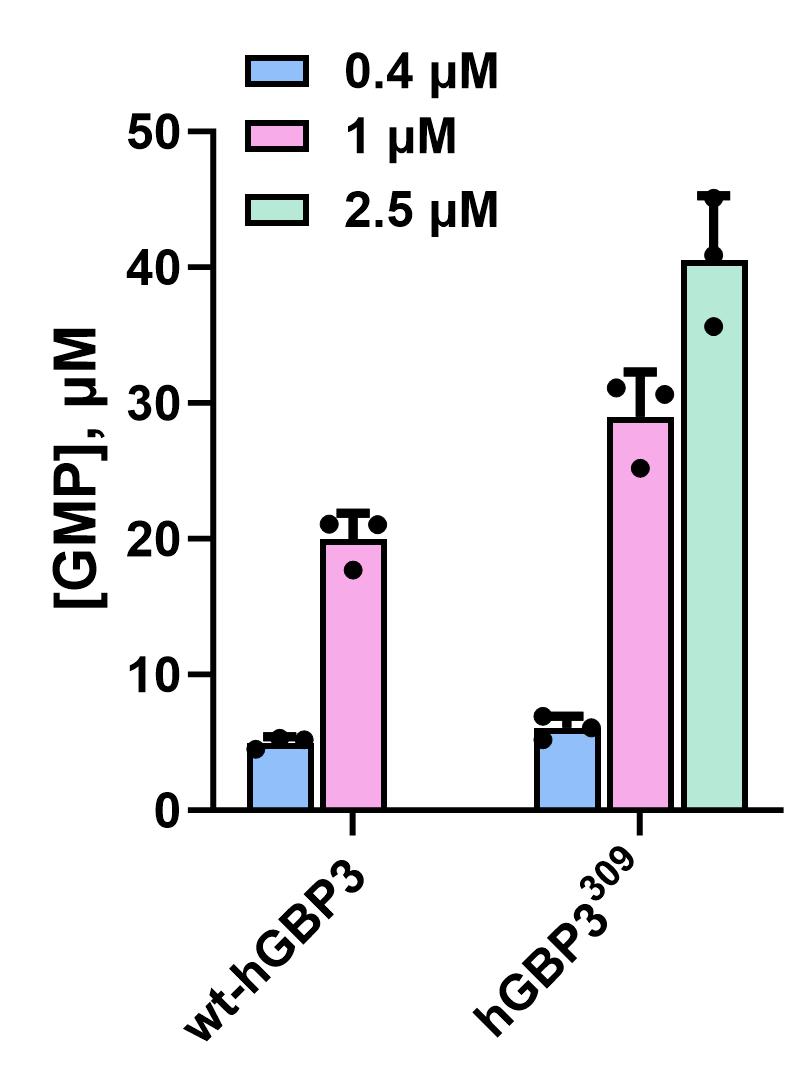

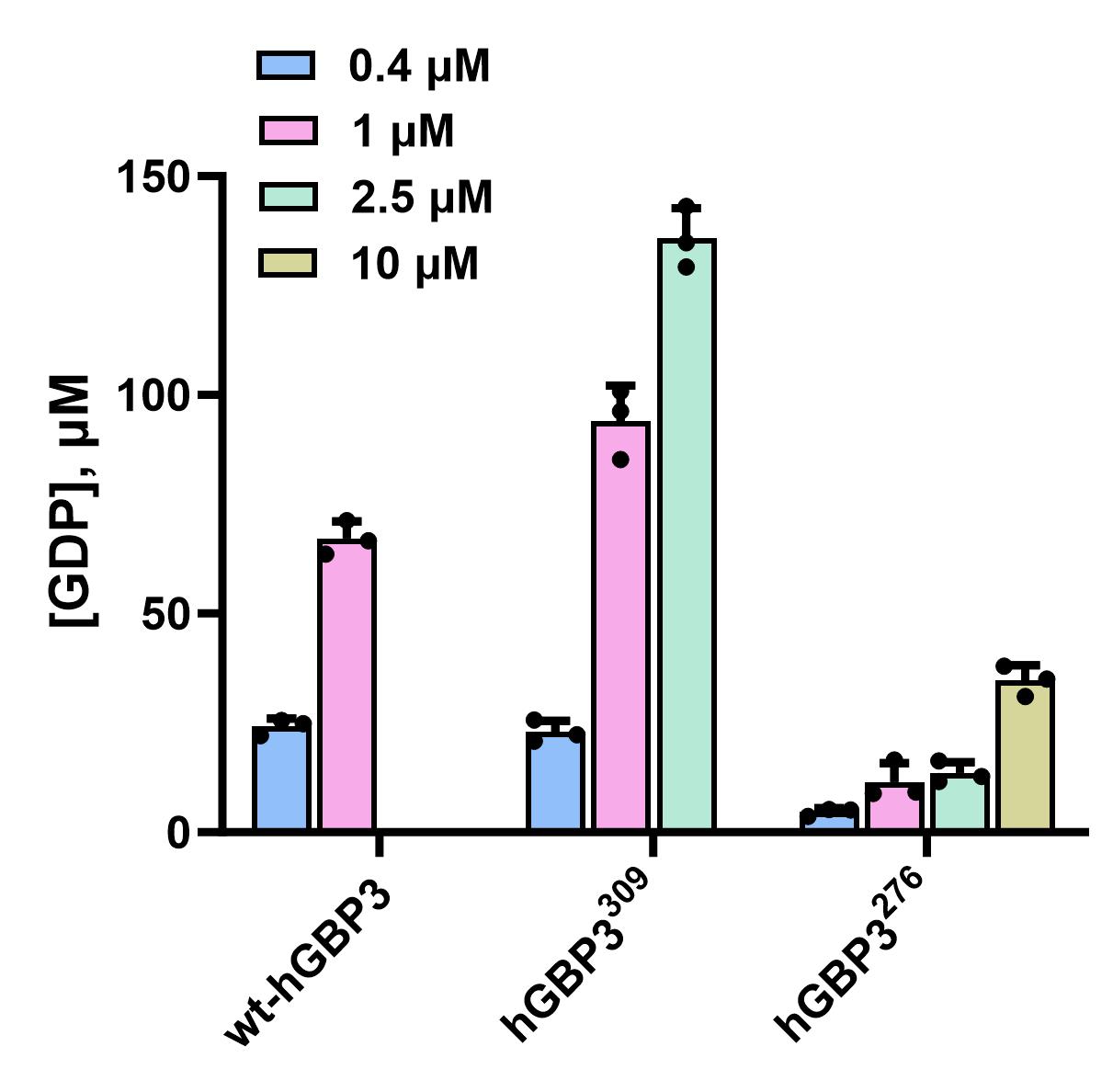


**A**

**B**

**Figure S2**. Bar graph showing the comparison of GDP (A) and GMP (B) production between full-length hGBP3 and its truncated variants, hGBP3^309^ and hGBP3^276^.


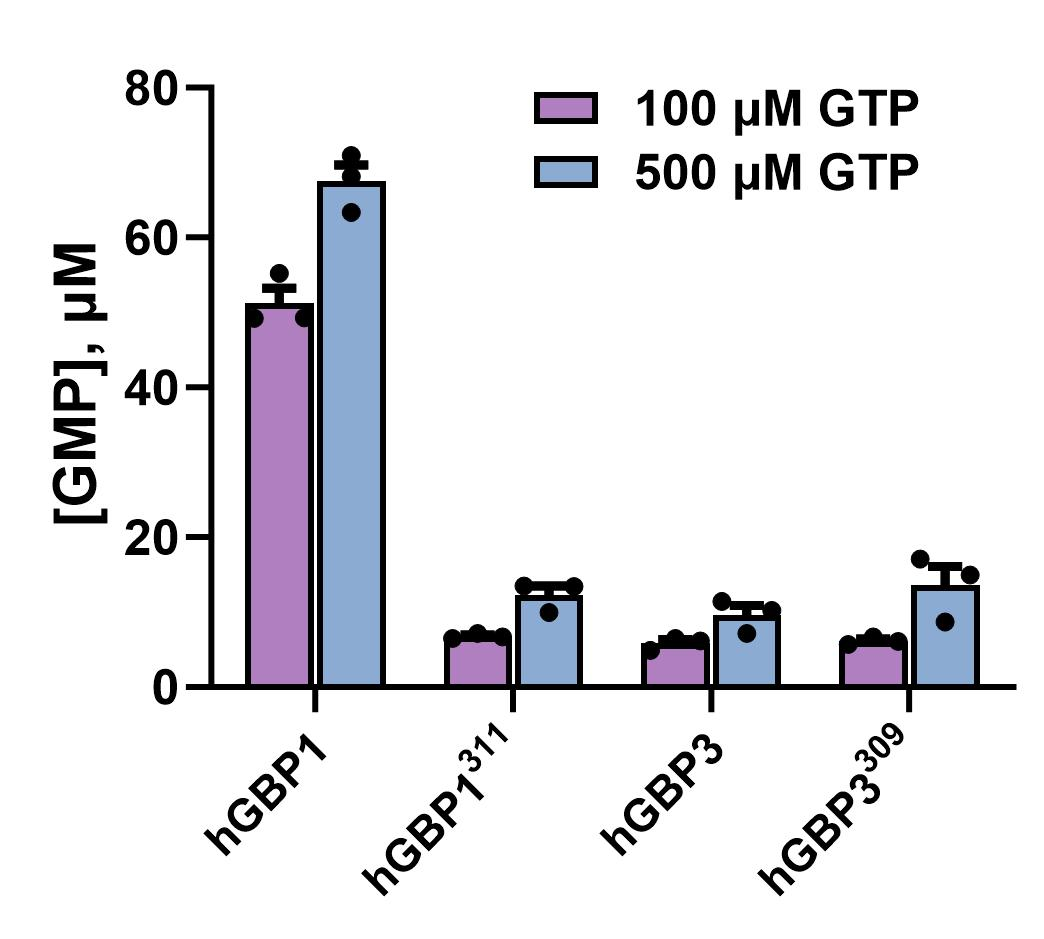

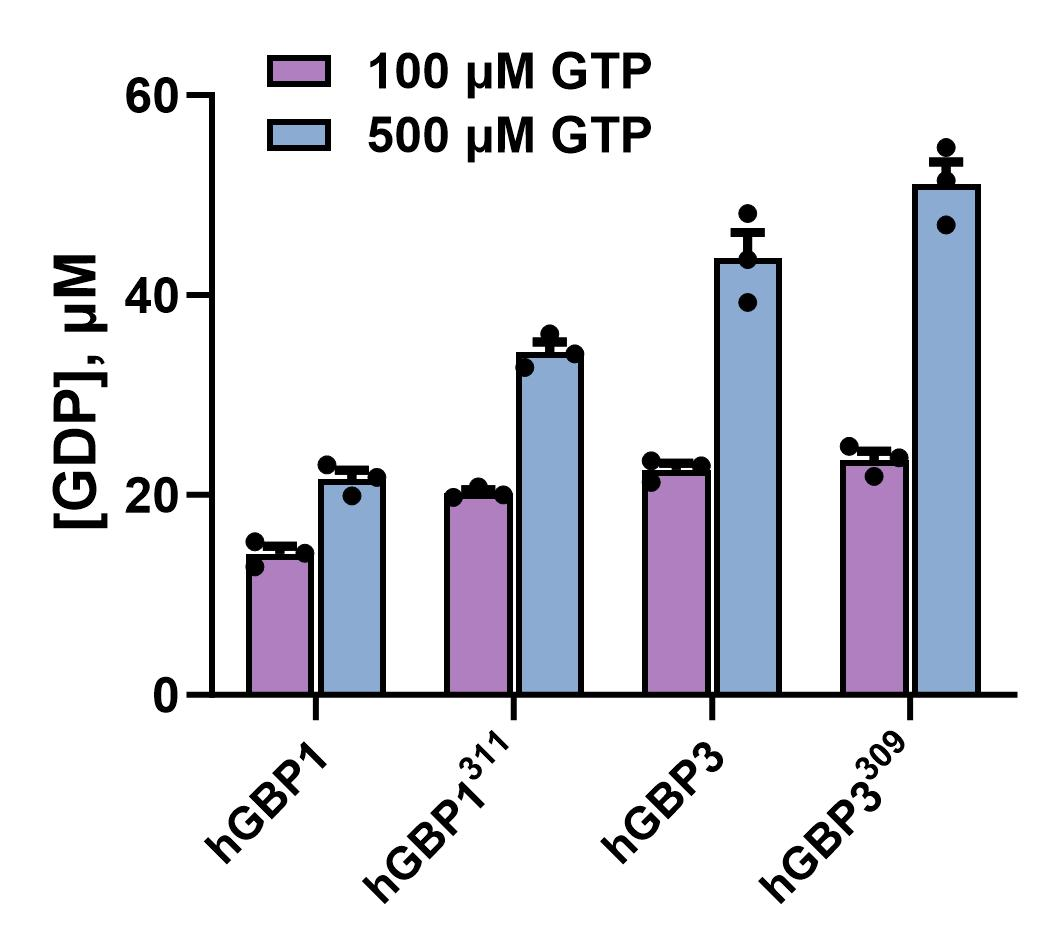


**Figure S3.** Comparison of the effect of helical domain deletion on GMP formation between hGBP3 and hGBP1. (A) GTPase activity assay with full-length and truncated variants of hGBP1 and hGBP3, hGBP1^311^ and hGBP3^309^. (B, C) The bar graphs representation of average GDP and GMP production, respectively, based on the results obtained from the activity assays.


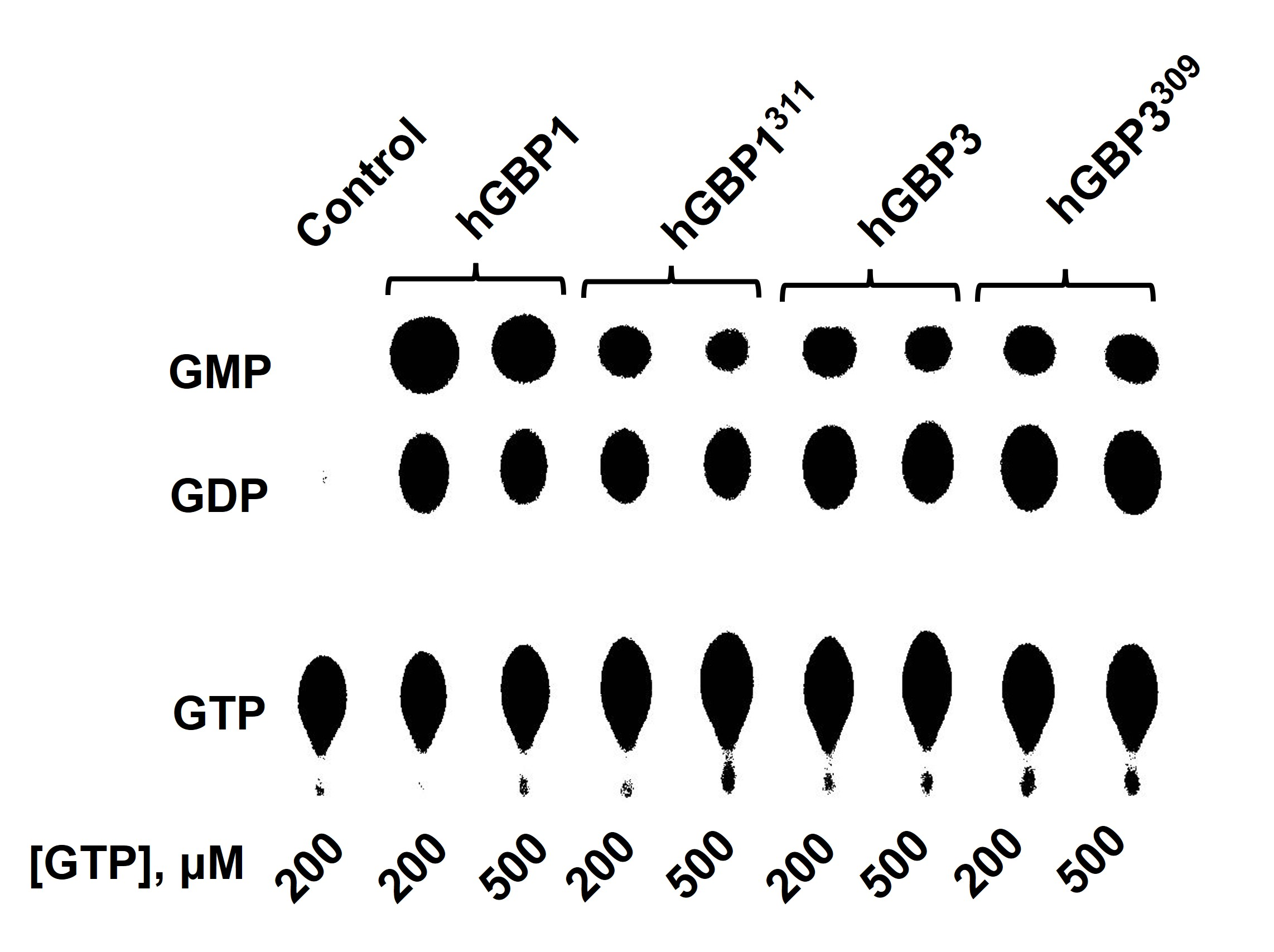


**A**

**C**

**B**


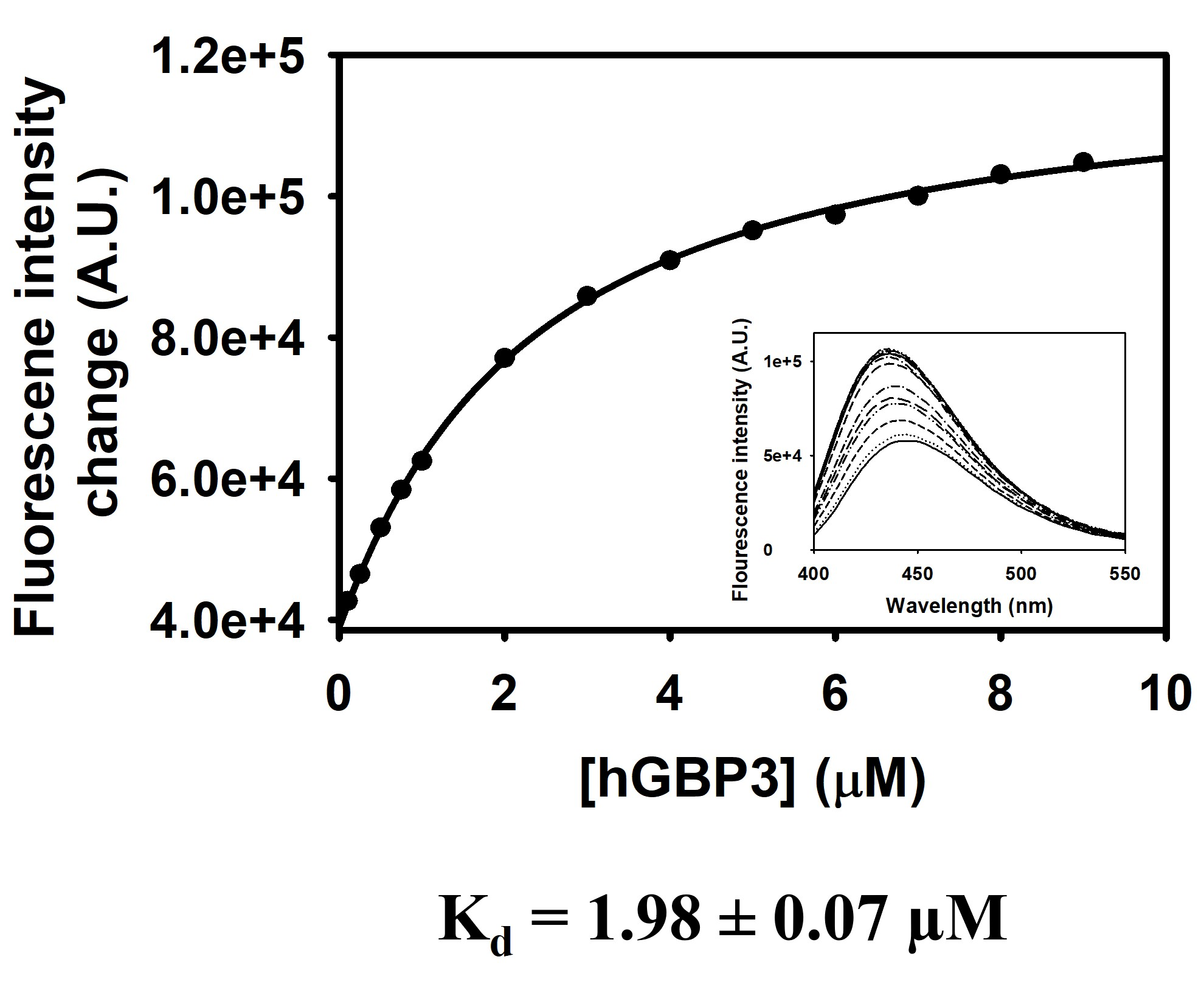


**Figure S4.** Representative plot of substrate affinity determination for hGBP3. Protein was titrated against fluorescently labeled GTP analog, mant-GppNHp. 366nm wavelength was used to excite the sample and the emission spectra were recorded in the range of 410 to 540nm. Fluorescence intensity at 435nm was plotted against the protein concentration. A quadratic equation (shown in the material method) was used to fit the data, which yielded the *K*_d_ value.


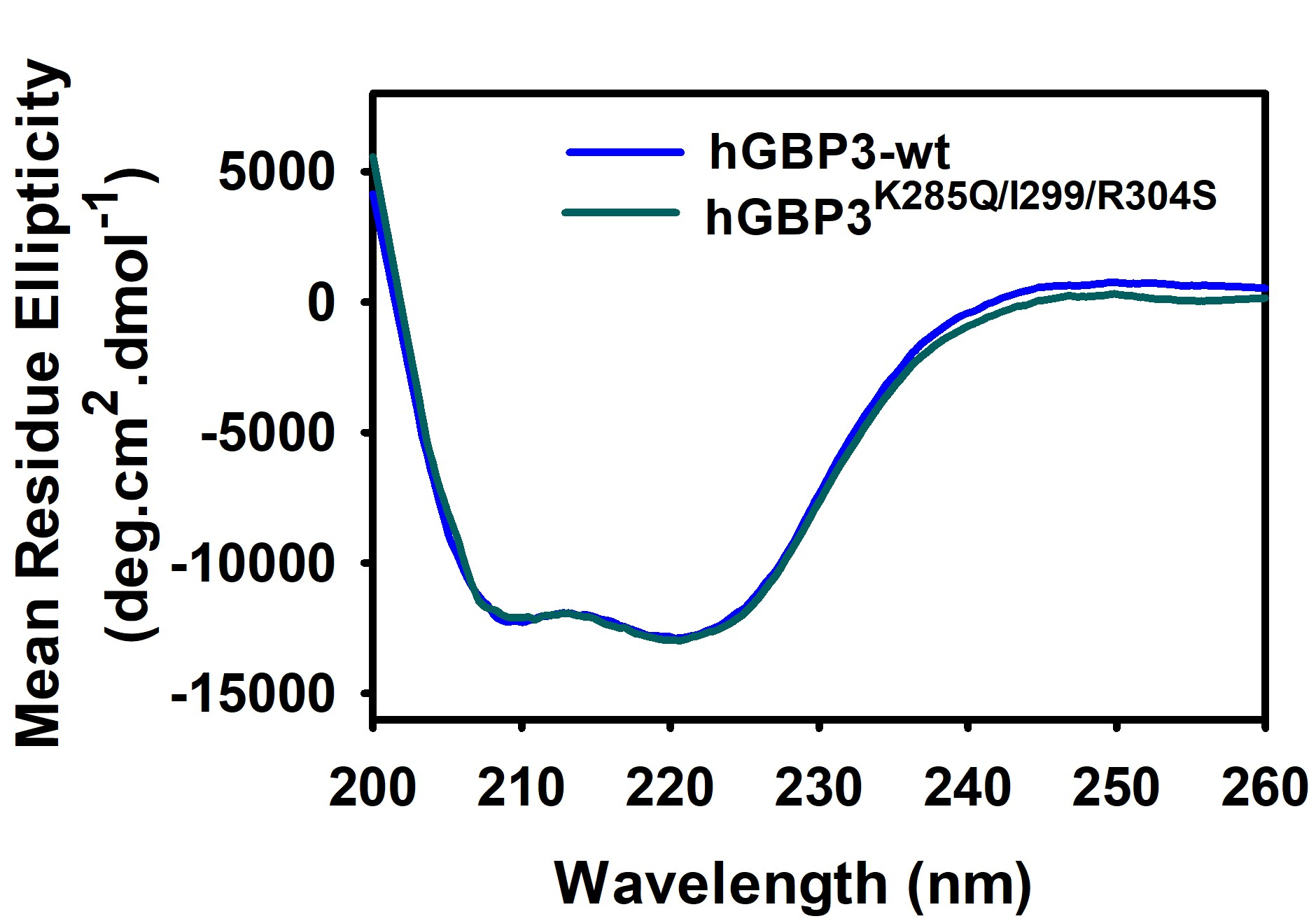


**Figure S5.** Secondary structure measurement of wild-type hGBP3 and its intermediate region triple mutant hGBP3^K285Q/I299V/R304S^.


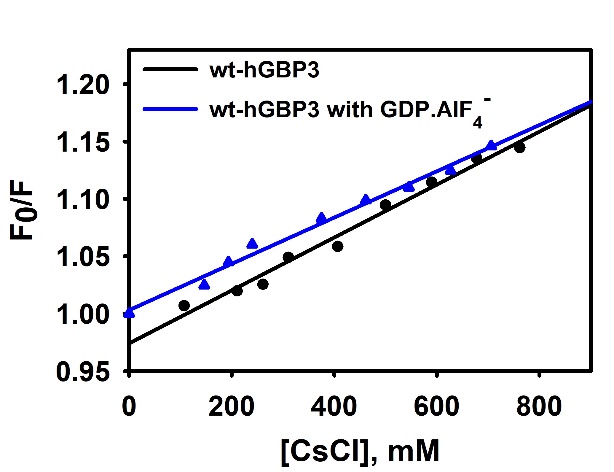

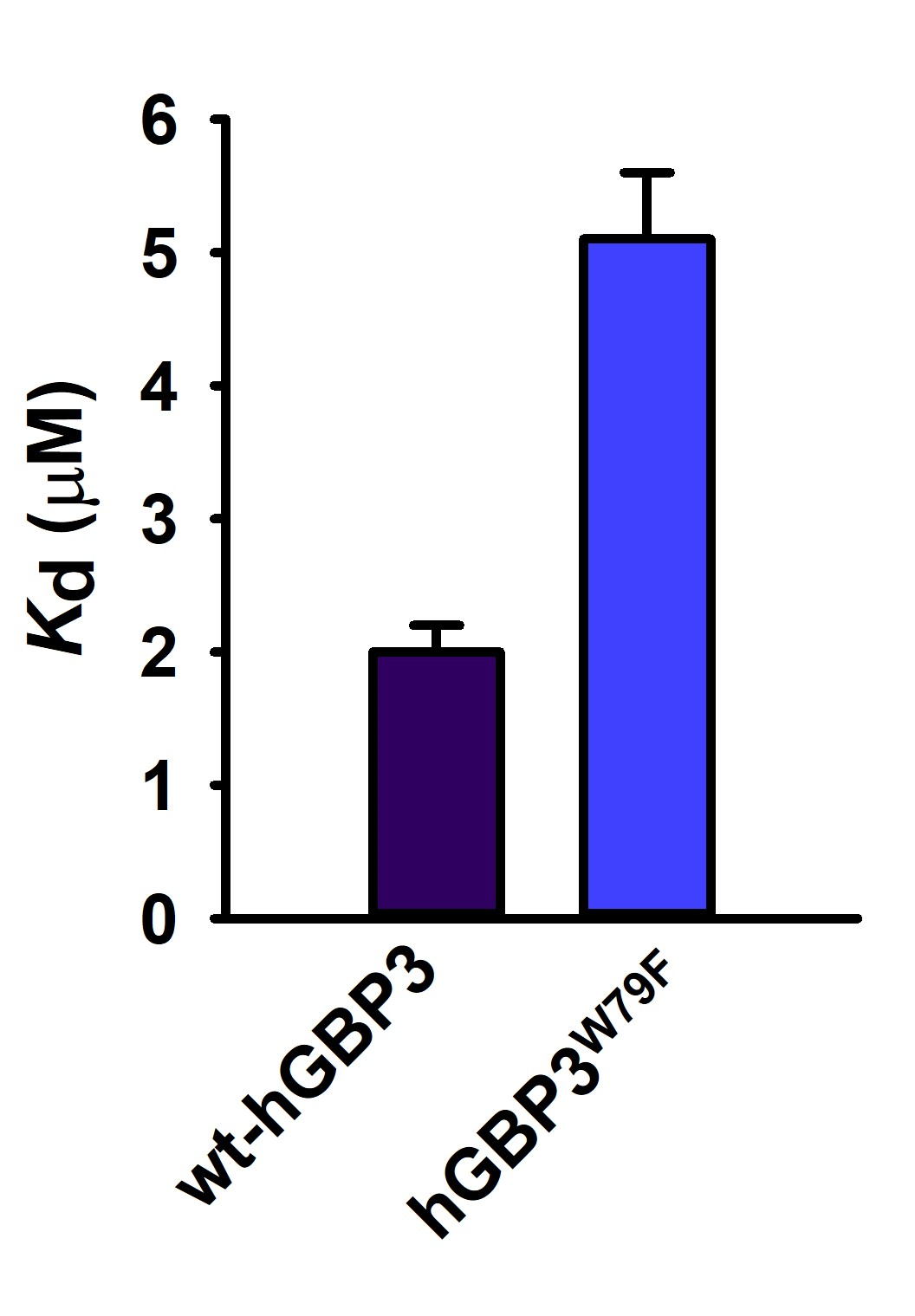


**Figure S6**. Comparison of *K*_d_ value between wild-type hGBP3 and its W79F mutant. The *K*_d_ value of the mutant is ~2.5 times higher than the wild-type protein. However, it is unlikely to affect the protein’s activity as the assays were performed at 200 µm GTP concentration (99% of the enzyme were in complex with the substrate at activity assay conditions).

**A**

**B**

**Figure S7.** Dynamic tryptophan fluorescence quenching experiment with wild-type hGBP3 (A) and W79F mutant (B), in the presence and absence of GDP.AlF_4_^-^. Both graphs represent the titration of proteins with CsCl as a quencher. The fluorescence intensity at the emission maximum was measured and the Stern-Volmer plot (F_0_/F vs concentration of CsCl) was generated. The slope of the curve gave the value of Ksv.


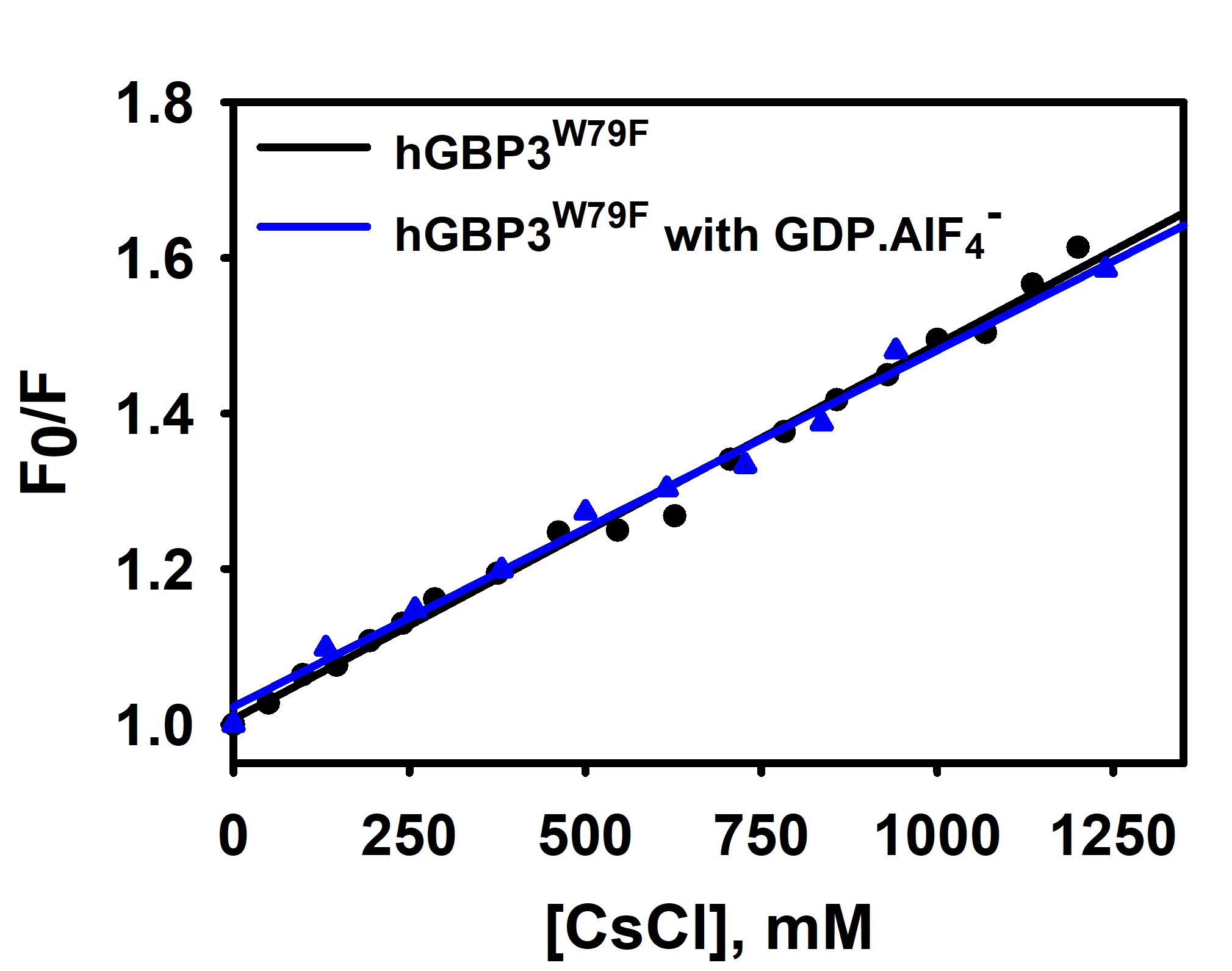

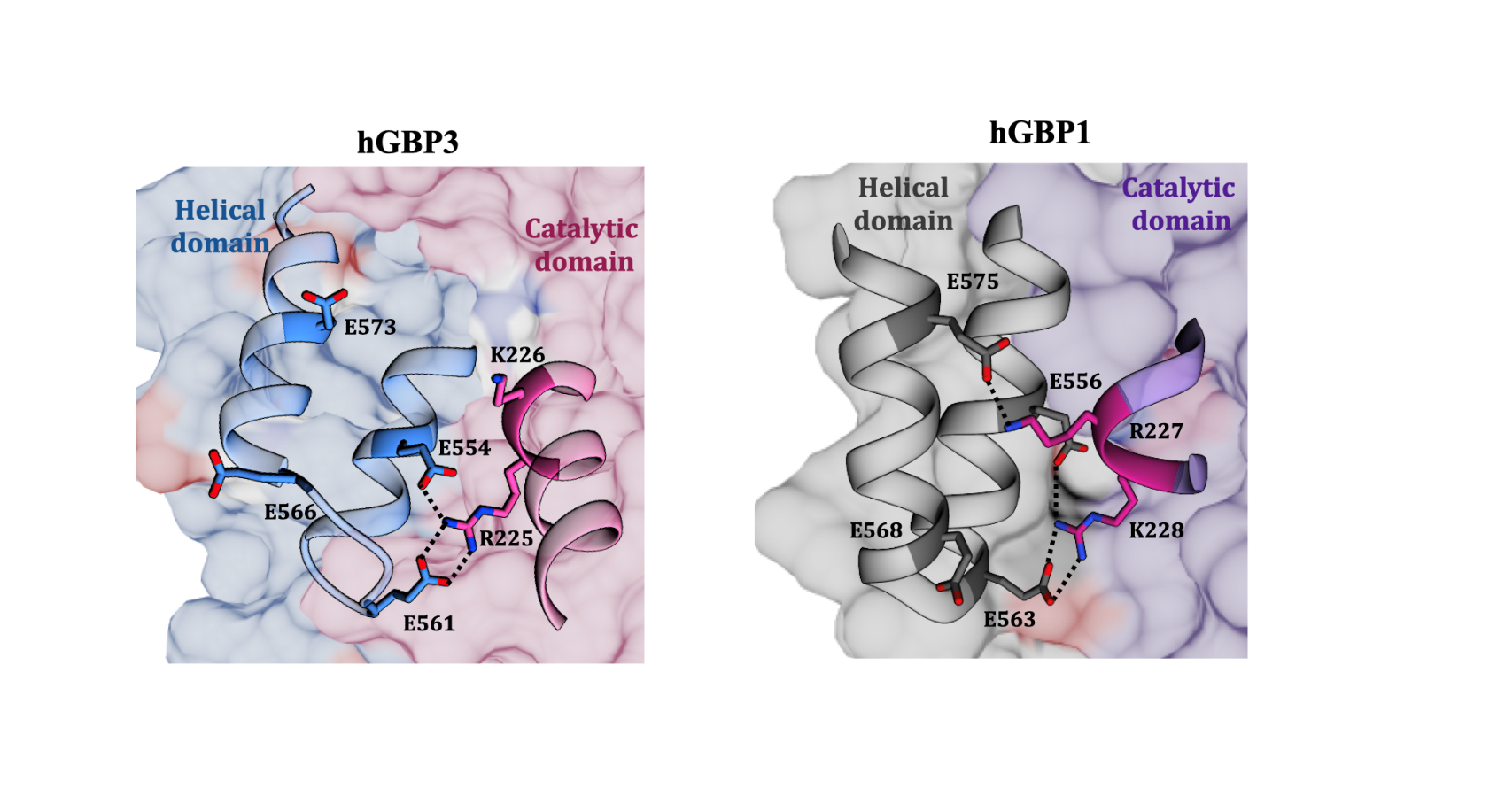


**Figure S8.** Inter-domain interactions between the catalytic and helical domains of (A) hGBP3 and (B) hGBP1.

**A**

**B**

**Figure S9**. Comparison of helices in the helical domains of hGBP3 (magenta) and hGBP1 (sky blue). The orientation and arrangement of helix 9, 11, 12 and 13 show variation as compared to that of hGBP1.


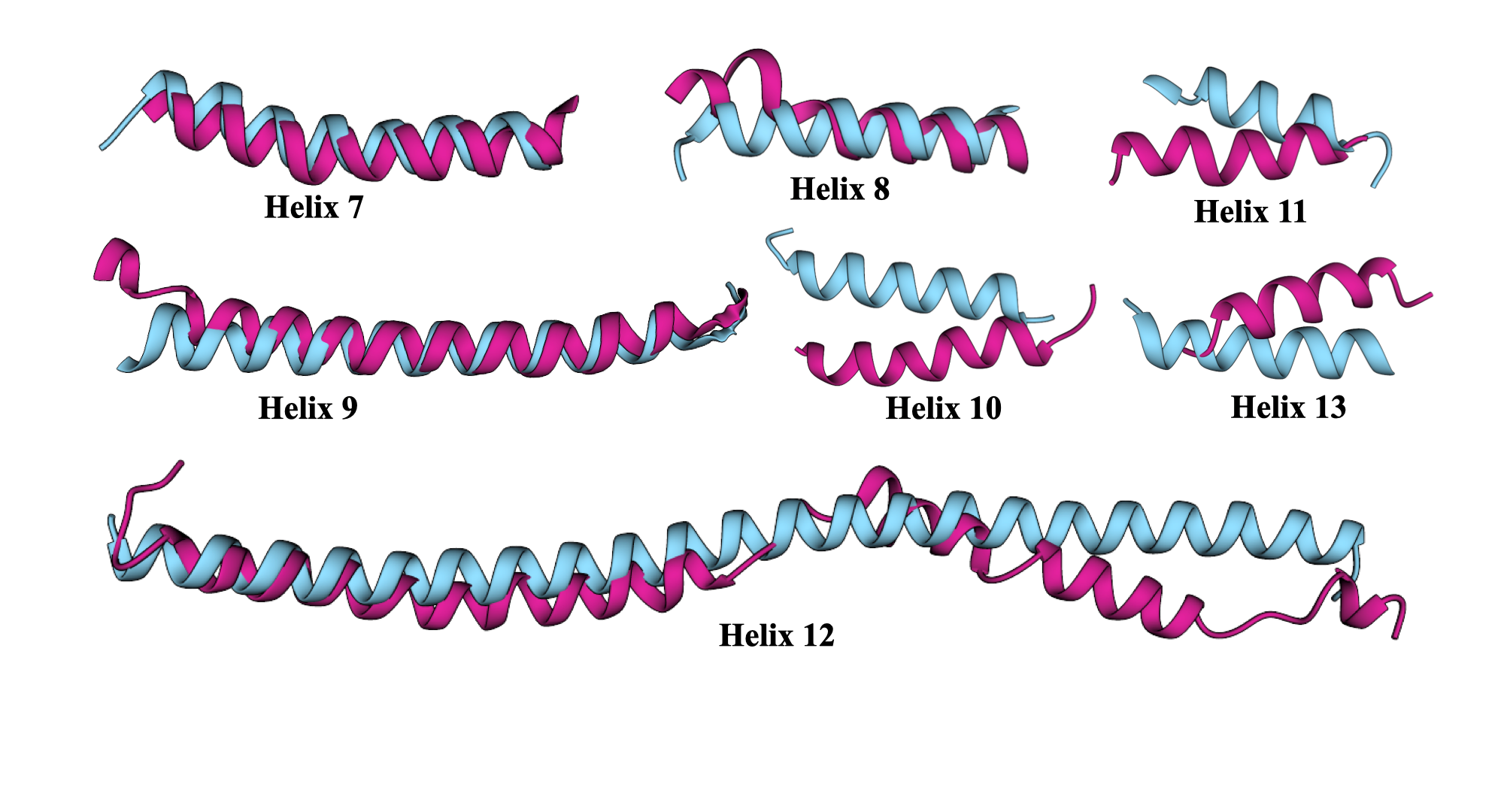

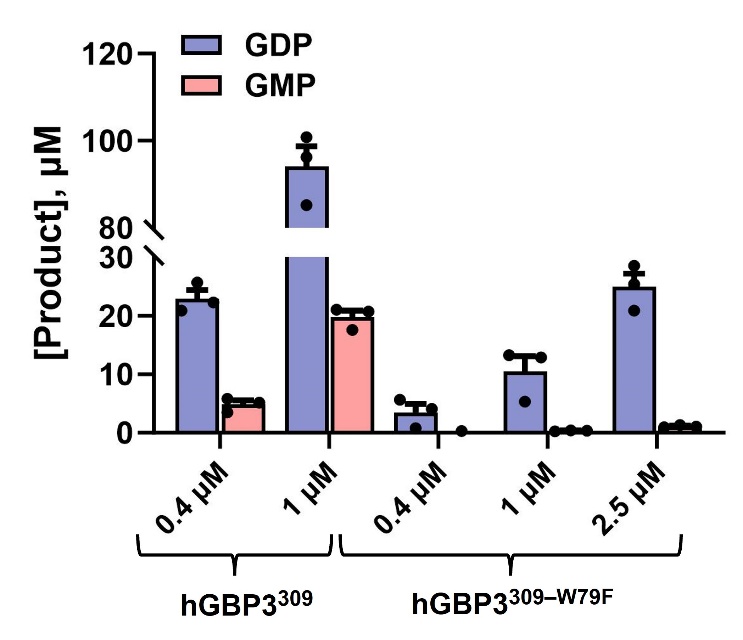

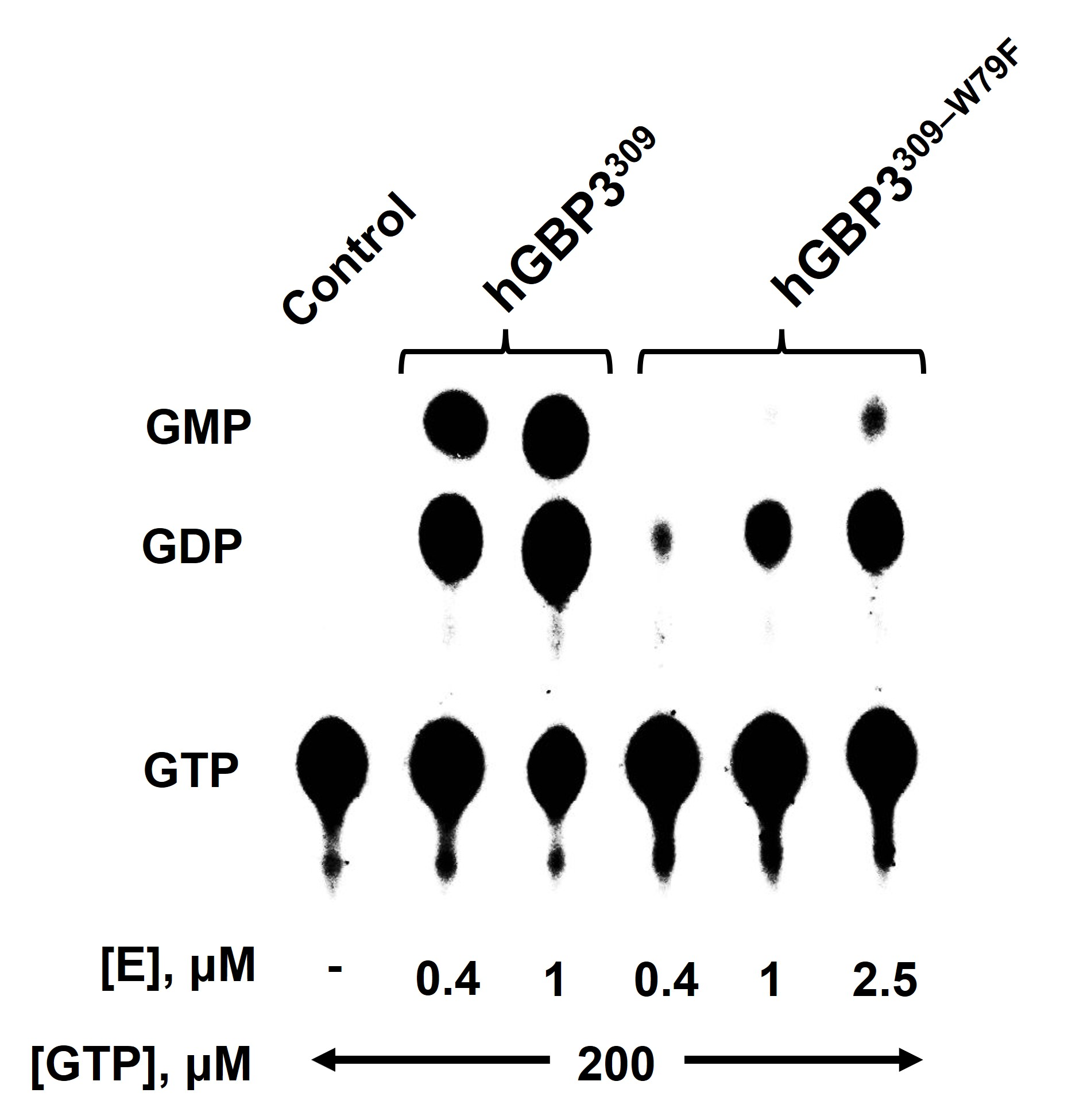


**A**

**B**

**C**

**D**

**Figure S10**. Helical domain does not influence the H-bond formation between indole N of W79 and carbonyl oxygen of K76. (A) GTPase activity assay with hGBP3 truncated variant hGBP3^309^ and its mutant hGBP3^309-W79F,^ (B) Bar graph representation of the data obtained from activity assay experiments. (C) and (D) Intrinsic tryptophan fluorescence measurements of hGBP3^309^ and hGBP3^309-W79F^ in the presence and absence of GDP.AlF_4_^-^_._


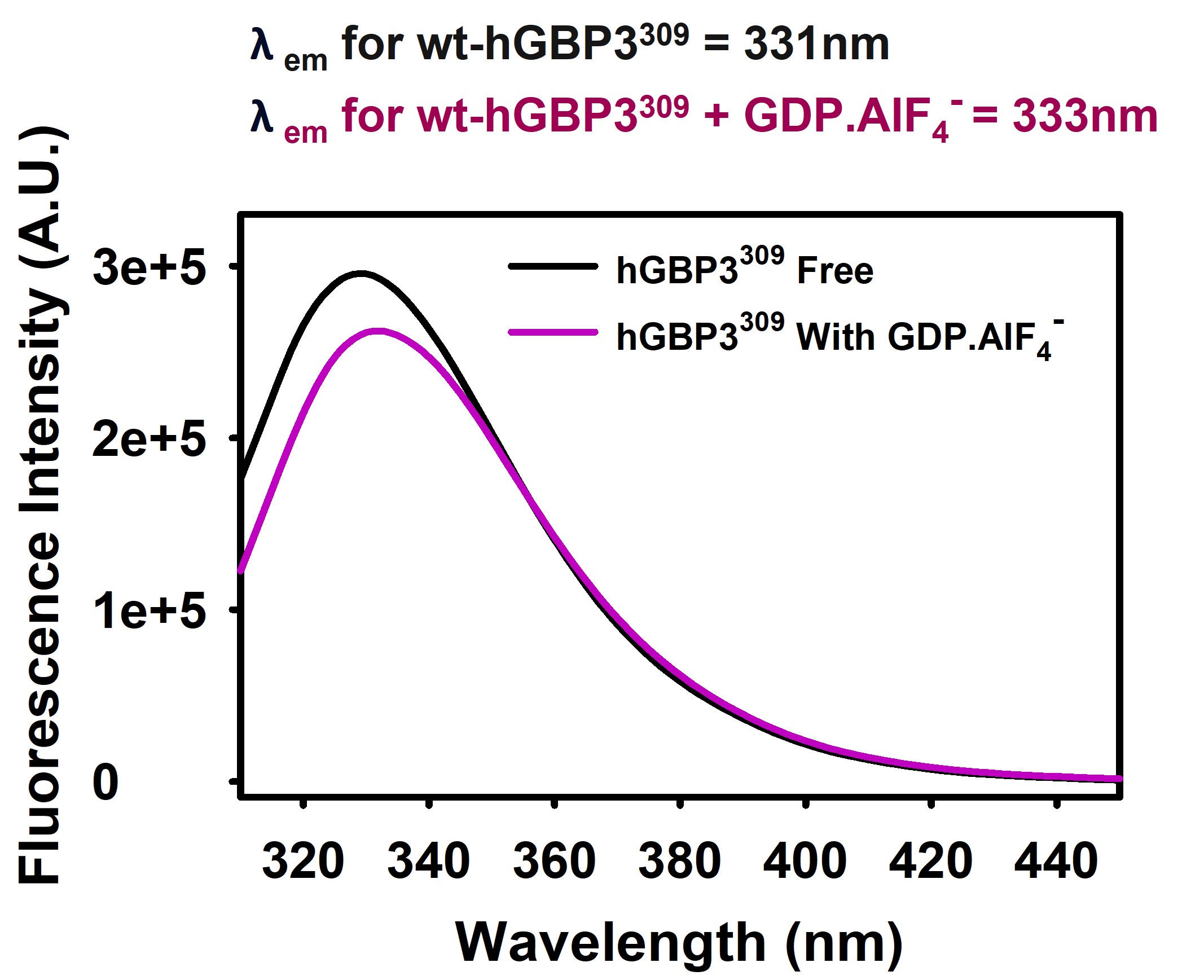

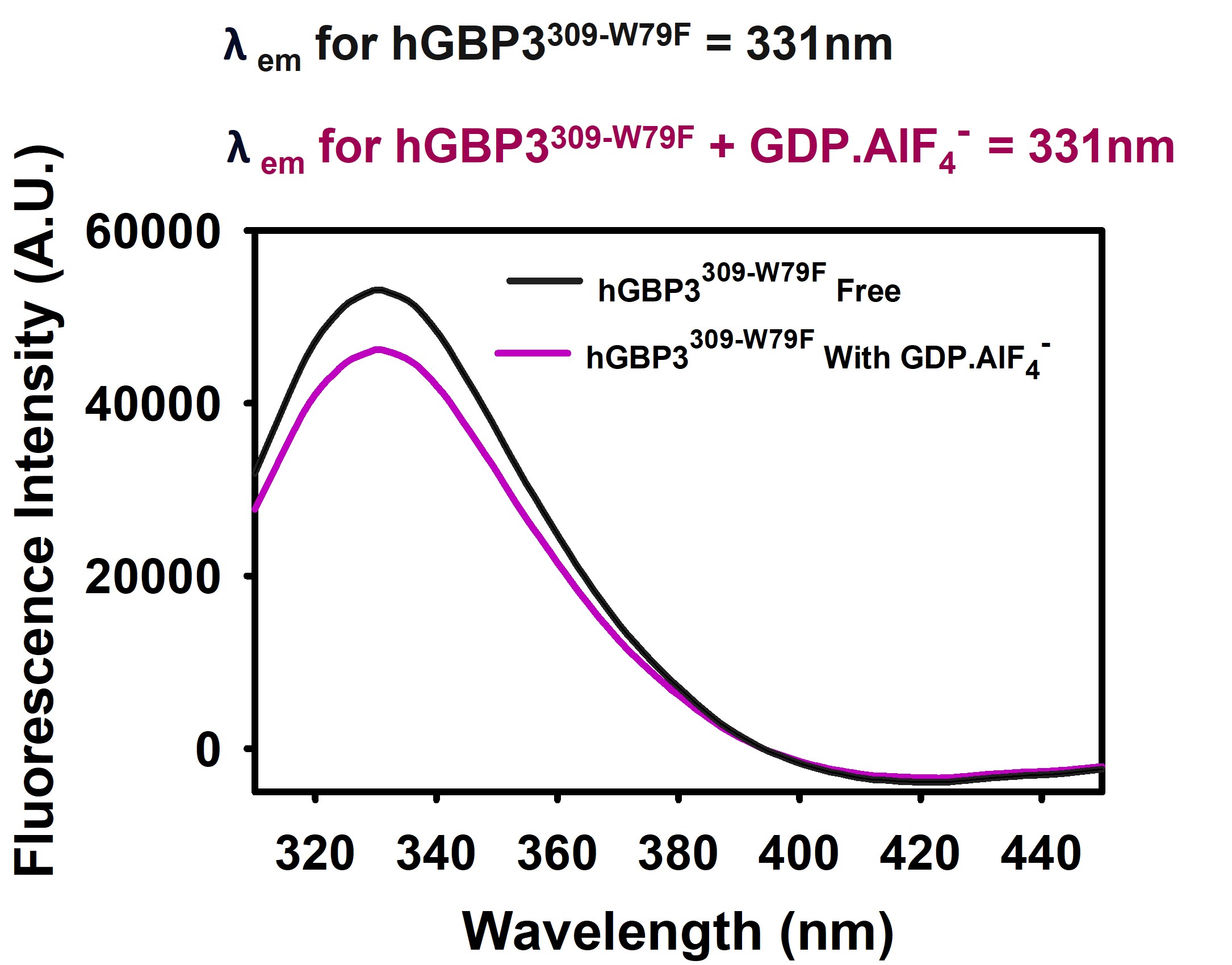

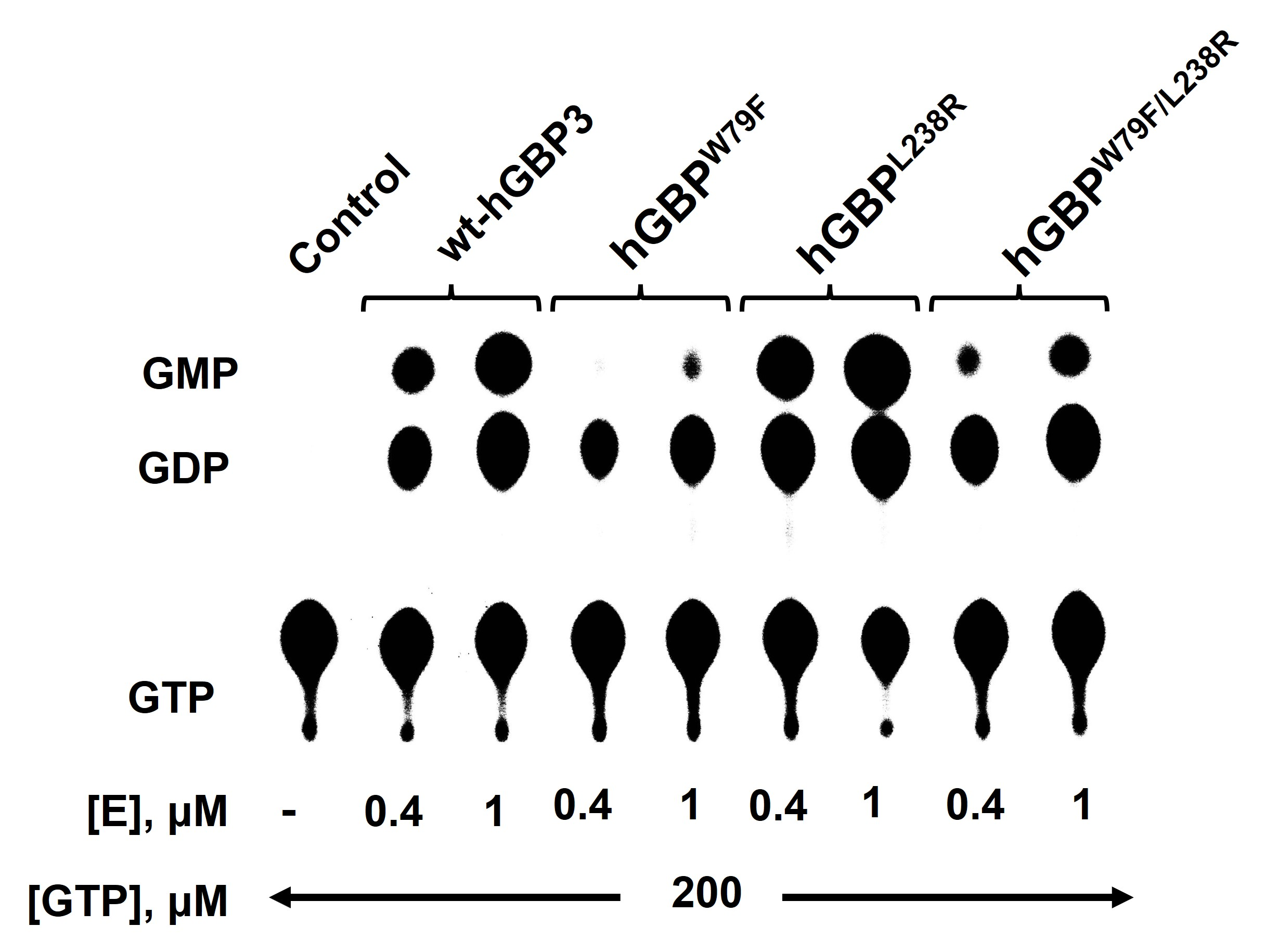


**A**

**B**

**Figure S11**. Guanine-cap mutation increases GMP by 10%. (A) GTPase assay with wt-hGBP3, hGBP3^W79F^, hGBP3^L338R^ and the double mutant hGBP3^W79F/L238R^ where the H-bond formation between indole N of W79 and carbonyl of K76 was hampered but the G-cap defect was primarily fixed. (B) Bar graph representation of the data obtained from activity assay experiments.


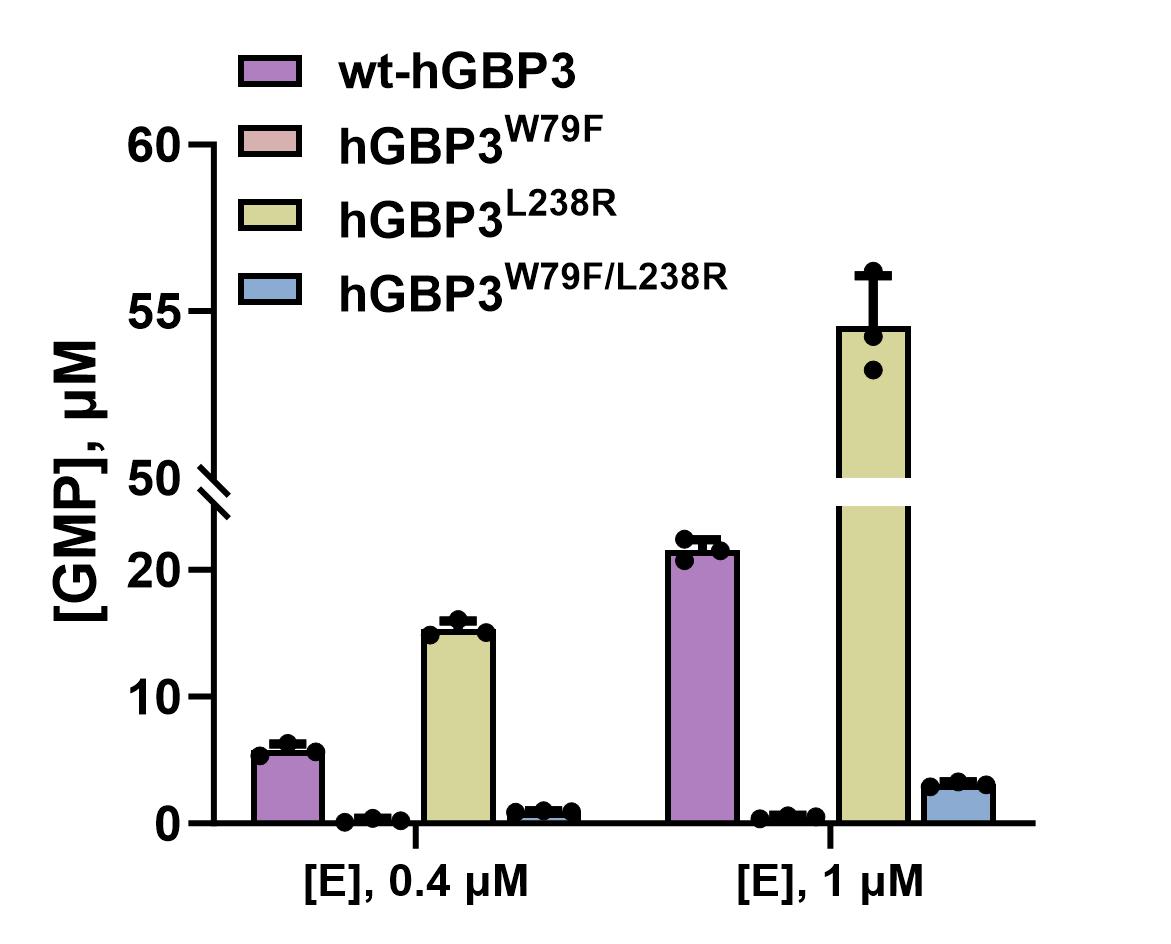


**Figure S12**. Ramachandran plot for the model structure of hGBP3 with GppNHp.


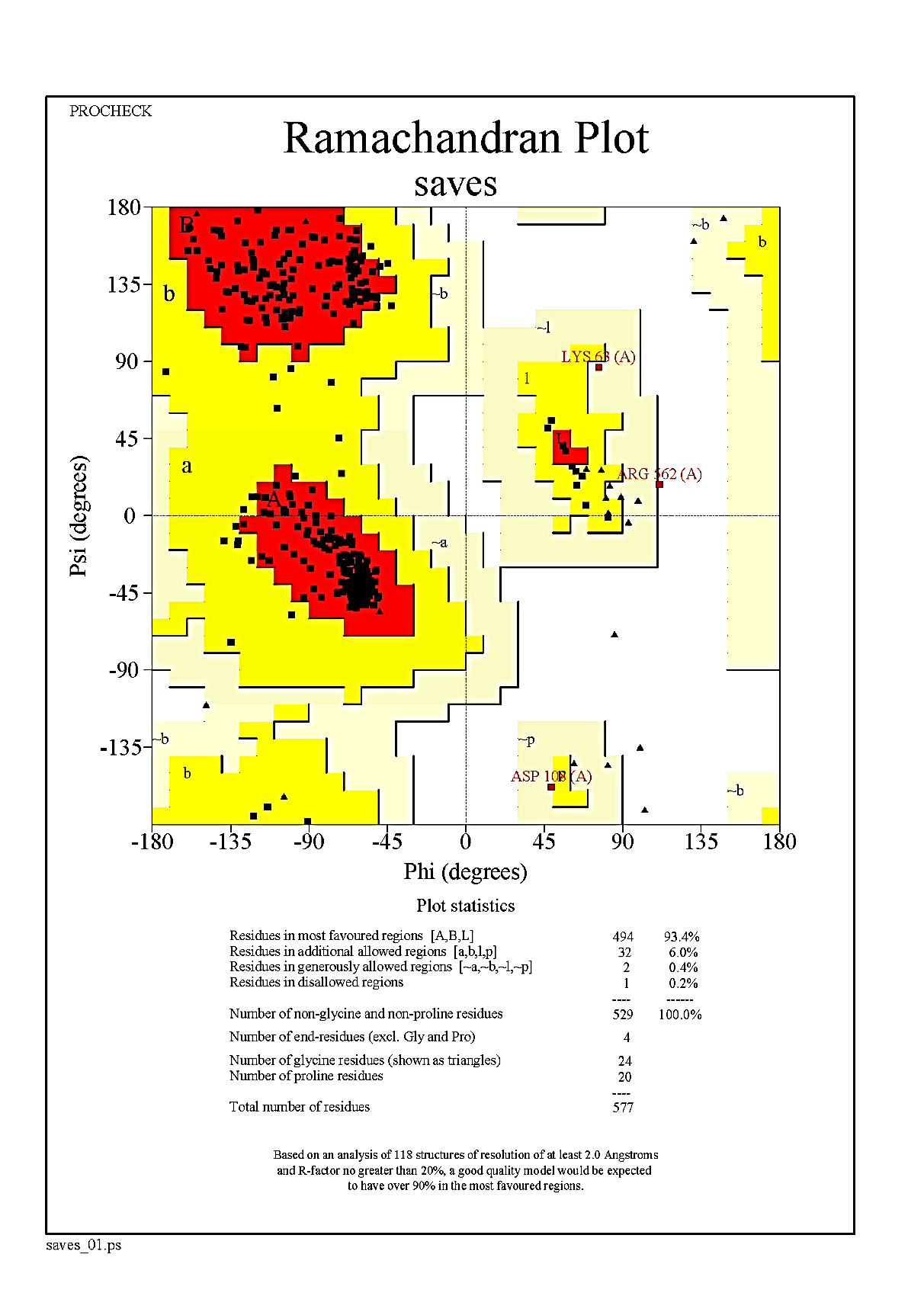

Supplement: Supporting Figures and Tables [file mmc1.docx]
